# A fat-derived metabolite regulates a peptidergic feeding circuit in *Drosophila*

**DOI:** 10.1371/journal.pbio.2000532

**Published:** 2017-03-28

**Authors:** Do-Hyoung Kim, Minjung Shin, Sung-Hwan Jung, Young-Joon Kim, Walton D. Jones

**Affiliations:** 1 Department of Biological Sciences, Korea Advanced Institute of Science and Technology (KAIST), Daejeon, South Korea; 2 School of Life Sciences, Gwangju Institute of Science and Technology (GIST), Gwangju, South Korea; The Francis Crick Institute, United Kingdom

## Abstract

Here, we show that the enzymatic cofactor tetrahydrobiopterin (BH4) inhibits feeding in *Drosophila*. BH4 biosynthesis requires the sequential action of the conserved enzymes Punch, Purple, and Sepiapterin Reductase (Sptr). Although we observe increased feeding upon loss of Punch and Purple in the adult fat body, loss of Sptr must occur in the brain. We found Sptr expression is required in four adult neurons that express neuropeptide F (NPF), the fly homologue of the vertebrate appetite regulator neuropeptide Y (NPY). As expected, feeding flies BH4 rescues the loss of Punch and Purple in the fat body and the loss of Sptr in NPF neurons. Mechanistically, we found BH4 deficiency reduces NPF staining, likely by promoting its release, while excess BH4 increases NPF accumulation without altering its expression. We thus show that, because of its physically distributed biosynthesis, BH4 acts as a fat-derived signal that induces satiety by inhibiting the activity of the NPF neurons.

## Author summary

As the primary site of energy storage, adipose tissue must somehow monitor energy reserves and communicate this information to the brain. The brain must then modulate feeding behavior to maintain energy balance; however, the mechanisms underlying this communication between fat cells and the brain remain poorly understood. Here, we perform a targeted genetic screen in *Drosophila melanogaster* and identify a role for the enzymatic cofactor tetrahydrobiopterin (BH4) in regulating ad libitum feeding behavior in fruit flies. We show that three highly conserved enzymes—Punch, Purple, and Sepiapterin Reductase (Sptr)—are required for the biosynthesis of BH4. Fat body-specific knock-down of either Punch or Purple increases feeding, and this increase can be rescued by BH4. We find that rather than also being required in the fat body, Sptr is required in brain neurons that express neuropeptide F (NPF), the fly homologue of the vertebrate appetite regulator neuropeptide Y (NPY). BH4 also rescues the increase in feeding caused by NPF neuron-specific knock-down of Sptr. Although the exact mechanism remains unclear, our results suggest that BH4 inhibits signaling through NPF neurons by blocking their release of NPF. Based on its novel function in feeding and its physically distributed biosynthesis, BH4 may, therefore, represent one of the elusive signals that communicates energy status from the adipose tissue to the brain.

## Introduction

Animals must balance food intake with energy expenditure to maintain optimal health. In choosing what and how much to eat, animals integrate external cues like tastes and smells with internal motivational states like hunger and satiety. Powerful homeostatic mechanisms tie these motivational states to the sensing of nutrient and energy status. Because fat is the primary long-term energy storage molecule, these homeostatic sensors monitor fat levels—triggering increased feeding when they fall and decreased feeding when they rise. In mammals, adipocytes secrete leptin, which then circulates to the brain to reduce appetite via leptin receptors in key hypothalamic nuclei [1]. The discovery and cloning of leptin [2] and the leptin receptor [3] were widely hoped to provide a solution to the problem of obesity. Although leptin replacement does dramatically reduce leptin-deficient obesity [4], the results of clinical trials assessing the ability of recombinant leptin to reduce body weight in patients with normal levels of leptin have been underwhelming [5]. This asymmetric action of leptin, seeming primarily to signal fat loss rather than tracking levels of both fat loss and fat gain, is one piece of evidence suggesting the existence of other unknown fat-derived signals that tell the brain when fat stores are large enough to reduce feeding [6].

*D*. *melanogaster* are emerging as a powerful model system for studying the molecular mechanisms of metabolism and appetite regulation because they couple rapid development and a wealth of tools for manipulating both the genome and the activity of neural circuits. Adult flies regulate food intake according to nutritional status and circadian rhythms like humans do [7,8]. They also have clear homologues for most of the human genes implicated in metabolic homeostasis and feeding [9]. The fat body-derived cytokine Unpaired-2 (Upd2), for example, acts indirectly on the fly insulin-producing cells to increase insulin production and release. Incredibly, mis-expression of human leptin rescues the *upd2*-null mutant phenotype, underscoring the similarity of the two systems [10].

Because there are aspects of the leptin system present even in flies, we decided to perform a two-tiered microRNA (miRNA)- and RNA interference (RNAi)-based genetic screen in *Drosophila* to identify novel fat-derived regulators of feeding. After finding in a primary screen that overexpression of *miR-iab-4* in the adult fat body enhances feeding, we performed a secondary screen of potential *miR-iab-4* targets expressed in the fat body. In this secondary screen, we found fat body-specific knock-down of *purple* (*pr*) also enhances feeding in adult flies. The *pr* locus encodes an enzyme, which, along with *Punch* (*Pu*) and *Sepiapterin reductase* (*Sptr*), functions in the synthesis of the enzymatic cofactor tetrahydrobiopterin (BH4). We confirmed Punch and Purple both function in the adult fat body to produce the intermediate 6-pyruvoyltetrahydropterin (PTP). This PTP seems then to circulate to the brain for conversion to BH4 by Sptr in neurons producing the feeding-related neuropeptide NPF. We show the increase in feeding caused by loss of Purple in the fat body is due to the loss of BH4 in NPF neurons. This loss seems, by an as yet unknown mechanism, to reduce the activity of the NPF neurons and inhibit their release of NPF, inducing satiety.

## Results

### Primary miRNA overexpression screen

**Fig 1:**
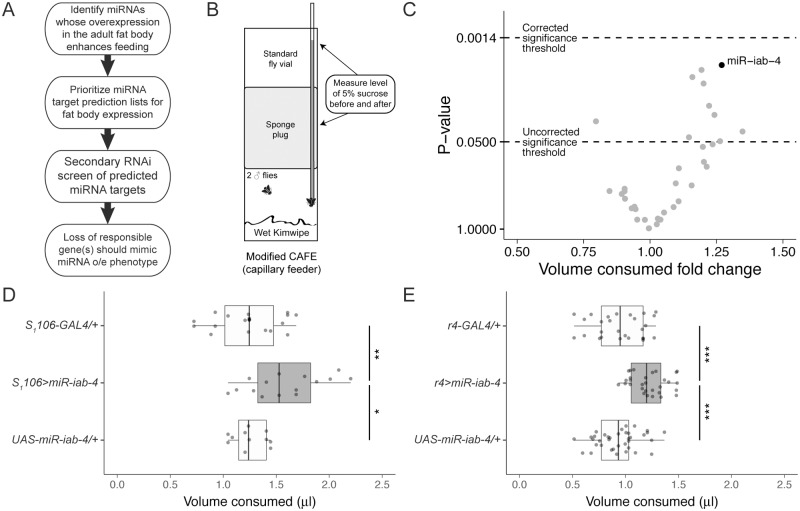
Primary genetic screen shows overexpression of *miR-iab-4* in the adult fat body increases feeding. **(A)** Flowchart explaining our two-tiered microRNA (miRNA)-based screen for fat body modulators of feeding. **(B)** Schematic of the modified capillary feeder (CAFE) assay. **(C)** Summary of the primary miRNA overexpression screen. Each dot represents the feeding of S_1_106-GAL4 driving the expression of one UAS-miRNA in the fat body. The feeding fold change versus the heterozygous *S*_*1*_*106-GAL4/+* control plotted against the *p* value as determined by a Student’s *t* test. Significance thresholds, both uncorrected and corrected for multiple comparisons (Bonferroni method), are indicated. See S1 Table for a complete list of UAS-miRNA lines with their feeding fold changes and resulting *p* values. See S1 Fig for a ranked bar plot showing the mean feeding for all lines in this primary screen. **(D** and **E)** Fat body-specific overexpression of *miR-iab-4* using **(D)** the RU486-inducible S_1_106-GAL4 (*n* = 11–20) and **(E)** the constitutive r4-GAL4 (*n* = 27–37), both in gray, increase feeding compared to heterozygous controls (white). Underlying numerical data for this figure can be found here: http://dx.doi.org/10.5061/dryad.8hm82.

To identify regulators of appetite expressed in the fat body, we designed a two-tiered genetic screen like the one described by Bhat and Jones [11]. Through this primary screen, we hoped to identify an miRNA or miRNAs whose tissue-specific overexpression in the fat body alters adult feeding. After limiting a list of predicted miRNA targets to those expressed in the fat body, we planned to perform a small secondary RNAi screen for a gene or genes responsible for the feeding phenotype (Fig 1A). In the primary screen, we crossed a subset of a library of upstream activation sequence (UAS)-miRNAs [12] to the RU486-inducible fat body-specific GAL4 driver S_1_106-GAL4 generated by Roman et al. [13]. We used a modified form of the capillary feeder (CAFE) assay originally described by Ja et al. [14] to measure total adult feeding for 32 h (Fig 1B). In this primary screen, we found some miRNAs that reduce feeding and others that enhance feeding by up to approximately 35%. None of the miRNAs enhanced feeding enough, however, to produce a *p* value that met the Bonferroni-corrected threshold for statistical significance (Fig 1C, S1 Fig, and S1 Table). Of these results, we decided to follow up on the adult fat body-specific overexpression of *miR-iab-4*, which increases adult ad libitum feeding by 27% (Fig 1D). Overexpression of *miR-iab-4* using the constitutive fat body-specific driver r4-GAL4 [15] produces a slightly larger and more significant increase in adult feeding beyond that of the appropriate heterozygous controls than overexpression with S_1_106-GAL4 (Fig 1E).

### Secondary screen for fat body inhibitors of feeding

**Fig 2:**
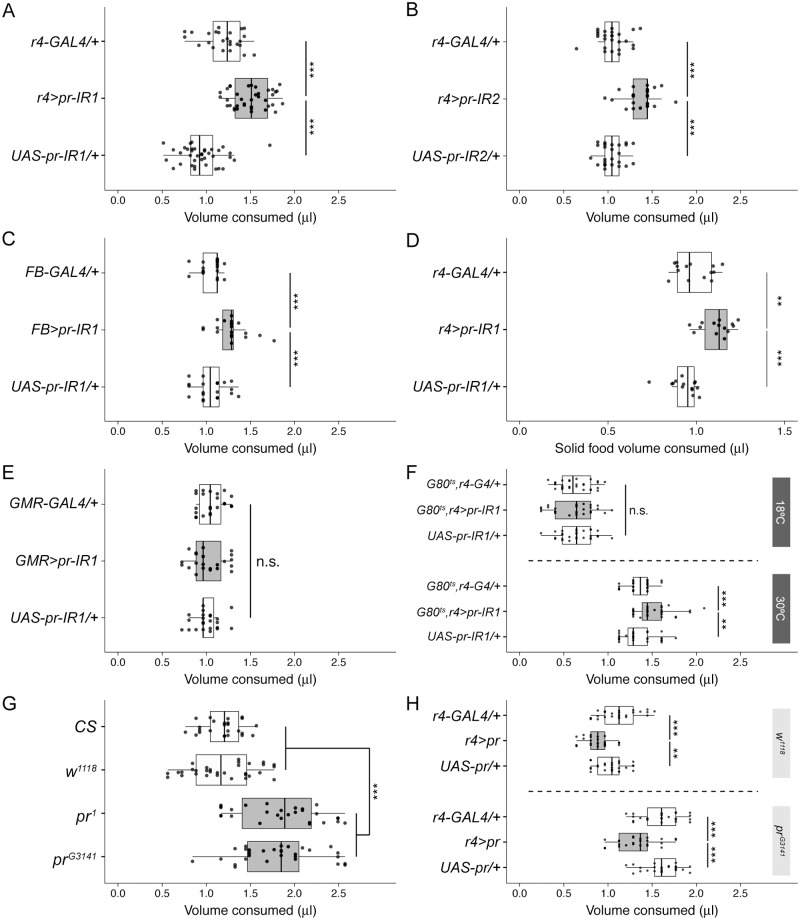
Purple expression in the fat body is required for feeding regulation. **(A)** Constitutive fat body-specific knock-down of *purple* (*pr*) using r4-GAL4 (*r4>pr-IR1*, gray boxplot) increases feeding (*n* = 25–38) when compared to heterozygous controls (white boxplots). See S2 Table for the rest of the secondary screen results. **(B)** Fat body-specific knock-down of *pr* with a second RNA interference (RNAi) line (UAS-pr-IR2) that targets a different part of *pr* also increases feeding (*n* = 25–26). **(C)** Knock-down of *pr* using a different fat body-specific GAL4 line (FB-Gal4) increases feeding (*n* = 20). **(D)** As with the capillary feeding (CAFE) assay, fat body-specific knock-down of *pr* increases the consumption of ^32^P-labeled solid food compared to the appropriate heterozygous controls (*n* = 14). **(E)** Eye-specific *pr* knock-down with GMR-GAL4 does not alter feeding (*n* = 23). **(F)** Adult fat body-specific knock-down of *pr* by combining r4-GAL4 with the temperature-sensitive tub-GAL80^ts^ increases feeding. Although there is no difference between genotypes at 18°C, *GAL80*^*ts*^,*r4>pr-IR1* flies transferred to 30°C post-eclosion eat more than heterozygous controls (*n* = 28–30). **(G)** Two hypomorphic *pr* mutants, *pr*^*1*^ and *pr*^*G3141*^ (gray boxplots), eat more than *Canton-S* and *w*^*1118*^ controls (white boxplots; *n* = 25–32). **(H)** Overexpression of *pr* in the fat body (gray boxplots) reduces feeding in the *w*^*1118*^ genetic background (above) and rescues the hyperphagia of the *pr*^*G3141*^ mutant background (below; *n* = 25). Underlying numerical data for this figure can be found here: http://dx.doi.org/10.5061/dryad.8hm82.

The relationship of a miRNA to its targets is sequence specific. Here, we are using miRNA overexpression as a tool not to explore the biology of the miRNAs themselves but to direct a follow-up screen using gene-specific RNAi. We thus compiled a list of potential *miR-iab-4* targets using the online prediction algorithm TargetScan [16]. Using the FlyAtlas tissue-specific expression data [17] available on FlyBase, we limited this list of potential *miR-iab-4* targets to those expressed in the adult fat body. In a small secondary screen using gene-specific RNAi (See S2 Table for a list of screened genes), we identified *pr* as a regulator of feeding behavior in *Drosophila*. When combined with the fat body driver r4-GAL4, we found two different pr-inverted repeat (IR) lines, each targeting distinct parts of the *pr* mRNA, that both enhance adult ad libitum feeding in the CAFE assay (UAS-pr-IR1, Fig 2A; UAS-pr-IR2, Fig 2B). Similarly, FB-GAL4 [18], another fat body-specific GAL4 line, also enhances adult feeding when combined with UAS-pr-IR1 (Fig 2C). Next, we were curious whether this increase in appetite is specific to the liquid sucrose-based food used in the CAFE assay or whether it also extends to standard solid media. When we performed the quantitative solid food feeding assay developed by Deshpande et al. [19], we found that *r4>pr-IR1* flies eat significantly more ^32^P-labeled standard cornmeal agar fly food than the relevant heterozygous controls (Fig 2D). This suggests *pr* loss-of-function in the fat body induces a general appetite enhancement rather than a craving for sucrose.

The *pr* gene got its name from one of its most recognizable mutant phenotypes—purplish eyes [20]. Indeed, according to FlyAtlas, the eye is the site of highest *pr* expression. There, *pr*, which encodes the enzyme PTP synthase (PTPS), is critical for the synthesis of the drosopterin class of light-screening eye pigments [21]. Using the eye-specific GMR-GAL4 to knock down *pr*, we found the expression of *pr* in the eye seems unrelated to feeding regulation (Fig 2E).

Although r4-GAL4 and FB-GAL4 are highly expressed in the adult fat body, they are also expressed in the larval fat body, making it possible the effect of *pr* on adult feeding we are observing could originate during development. Thus, we used the ubiquitously expressed temperature-sensitive tub-GAL80^ts^, which blocks the activity of GAL4 except at elevated temperatures, to limit our knock-down of *pr* to the adult fat body. At 18°C, the feeding of *tub-GAL80*^*ts*^;*r4-GAL4;UAS-pr-IR1* flies is indistinguishable from the appropriate heterozygous controls. After shifting them to 30°C post-eclosion so GAL80 can no longer block the GAL4-mediated expression of pr-IR1, flies of the same genotype eat more than heterozygous controls (Fig 2F). While there is a clear effect of ambient temperature on feeding amount, this result indicates *pr* in the adult fat body does influence feeding behavior.

To confirm this effect of *pr* on feeding behavior, we measured the feeding of two hypomorphic *pr* mutant strains (i.e., *pr*^*1*^ and *pr*^*G3141*^) and found both eat more than the “wild-type” controls *w*^*1118*^ and *Canton-S* (Fig 2G). Although we performed this experiment on male flies, we confirmed that *pr* mutant females also eat more than *w*^*1118*^ and *Canton-S* controls (S2 Fig). The increased feeding of the *pr* mutants also remained after seven generations of back-crossing to an isogenic *w*^*1118*^ strain (S3 Fig). We next asked whether fat body-specific overexpression of *pr* can rescue the hyperphagic phenotype of the *pr*^*G3141*^ mutant. In the absence of the *pr*^*G3141*^ mutation, transgenic overexpression of *pr* in the fat body produces a small but significant reduction in feeding compared to heterozygous controls (Fig 2H, above). In the presence of the *pr*^*G3141*^ mutation, *pr* overexpression produces a much more robust reduction in feeding compared to the appropriate controls (Fig 2H, below). Together, these data confirm normal feeding behavior requires fat body expression of *pr*.

Because body size and appetite typically show a strong positive correlation, we wondered whether loss of *pr* in the fat body may somehow induce growth, increasing body size and weight enough to account for the observed increase in feeding. Instead, we were surprised to find that 1-d-old *r4>pr-IR1* flies are slightly smaller than the heterozygous *r4-GAL4/+* control and similar to the *UAS-pr-IR1/+* control (S4A Fig). Because these flies also show a similar distribution of body weights (S4B Fig), we cannot attribute the increase in feeding induced by *pr* loss-of-function to enhanced body growth. Next, we reasoned that loss of *pr* may somehow increase locomotor activity and energy demand. This was not the case, as flies with *pr*-depleted fat bodies show levels of total activity (S4C Fig) and waking activity (S4D Fig) similar to the controls.

For further clues to the mechanism by which *pr* in the fat body affects feeding, we next asked whether the increase in feeding caused by *pr* loss-of-function also increases stored fat. Indeed, 15-d-old flies with *pr*-depleted fat bodies accumulate more triacylglycerides (TAGs) than controls (S5A Fig). We then measured lifespan of these flies in the absence of food to determine whether their excess fat is accessible; flies with larger fat stores should be more resistant to starvation. We found fat body-specific knock-down of *pr* enhances starvation resistance (S5B Fig), suggesting the increase in feeding associated with *pr* loss-of-function leads to a larger supply of stored but mobilizable energy.

### Appetite regulation requires BH4

**Fig 3:**
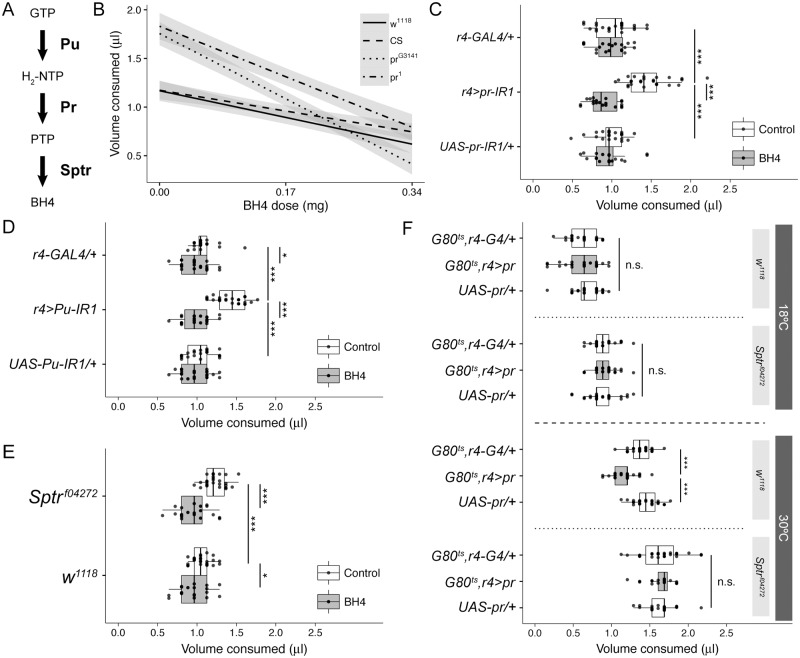
Purple regulates feeding via its role in BH4 biosynthesis. **(A)** Graphical summary of the BH4 biosynthesis pathway. GTP, guanosine triphosphate; H_2_-NTP, 7,8-dihydroneopterin triphosphate; PTP, 6-pyruvoyltetrahydropterin; BH4, 6R-L-erythro-5,6,7,8-tetrahydrobiopterin. **(B)** Feeding both wild-type and *pr* mutants BH4 causes a dose-dependent reduction in feeding (*n* = 20–40). The linear regression lines are shown in black, and their 95% confidence intervals are shaded in gray. Note the steeper decline in the hyperphagic *pr* mutants. **(C** and **D)** Pre-feeding with 0.17 mg/mL BH4 rescues the increase in feeding caused by fat body-specific knockdown of (**C**) *pr* (*n* = 20–26) and **(D)** *Punch* (*Pu*, *n* = 20–25). **(E)** Hypomorphic *Sptr*^*f04272*^ mutant flies eat more than *w*^*1118*^ controls, and their hyperphagia is rescued by pre-feeding with 0.17 mg/mL BH4 (*n* = 20–26). **(F)** While there is no effect at 18°C, at 30°C, adult fat body-specific overexpression of *purple* (*pr;* gray boxplots) reduces feeding below the level of the heterozygous controls (white boxplots) in the *w*^*1118*^ background but not the *Sptr*^*f04272*^ mutant background (*n* = 27). Underlying numerical data for this figure can be found here: http://dx.doi.org/10.5061/dryad.8hm82.

Because we confirmed normal appetite regulation requires *pr* in the fat body rather than in the eye, the feeding phenotype is unlikely related to the role the Purple protein plays in drosopterin eye pigment synthesis. Thus, we next explored the second known function of Purple as a catalyst for an intermediate step in the synthesis of the enzymatic cofactor BH4. In this pathway, Punch or guanosine triphosphate (GTP) cyclohydrolase converts GTP to 7,8-dihydroneopterin triphosphate (H_2_-NTP). Purple (PTPS) then converts H_2_-NTP to PTP, and Sptr converts the PTP to BH4 (Fig 3A) [22]. Since Kwak et al. showed oral BH4 can rescue the behavioral phenotypes (i.e., impaired motor function and dystonic hind-leg clasping) associated with Sptr loss-of-function mutations in mice [23], we converted the doses of BH4 effective in mice for use in *Drosophila*. Specifically, we chose two BH4 doses, one low (0.17 mg/mL) and one high (0.34 mg/mL). By adding BH4 to the 5% sucrose solution used to acclimate flies to the CAFE assay and switching back to pure 5% sucrose solution for the feeding assays, we can rule out any effect the taste of BH4 may have on the results. We found this BH4 pre-feeding produces a dose-dependent reduction in total feeding amount in all genotypes tested, but the effect is much stronger in the hypomorphic *pr* mutants *pr*^*1*^ and *pr*^*G3141*^ than in the “wild-type” controls *w*^*1118*^ and *Canton-S* (Fig 3B). Pre-feeding with the low BH4 dose also fully rescues the increase in feeding caused by fat body-specific knock-down of *pr* (Fig 3C).

Because we showed that hypomorphic *pr* mutants eat more than wild-type flies (Fig 2G), they may also be eating more during the BH4 pre-feeding stage. If so, they would be ingesting a higher dose of BH4 than the wild-type controls, invalidating any direct comparisons between the various strains for a given dose of BH4. We, therefore, performed a short-term feeding assay during the 24 h of BH4 pre-feeding by adding blue dye to the BH4-containing CAFE capillaries and taking pictures of the CAFE chambers every 4 h. This method allowed us to avoid disturbing the flies with our frequent measurements. As expected, we found that *pr*^*1*^ and *pr*^*G3141*^ eat faster than the *Canton-S* and *w*^*1118*^ controls, with the first statistically significant differences appearing 12 h into the assay. Neither *Canton-S* nor *w*^*1118*^ are affected when a low dose of BH4 (0.17 mg/mL) is added to the capillary fluid. In contrast, the same low dose of BH4 rescues the increased consumption of *pr*^*1*^ and *pr*^*G3141*^ flies to wild-type levels between 16 and 20 h into the assay (S5A and S5B Fig). Because the cumulative consumption of the *pr* mutants during low dose BH4 treatment drops to wild-type levels before the end of the 1 d pre-feeding period, direct comparisons between the *pr* mutants and wild-type controls in Fig 3B are valid.

Despite seeing no gross physical abnormalities or motor defects in BH4-treated flies, we remained concerned that BH4 may be reducing feeding simply because it might be toxic and makes flies too sick to feed. To address this, we fed *Canton-S* and *w*^*1118*^ flies continuously with high-dose BH4 (0.34 mg/mL) and measured their resulting lifespans. We found that high dose BH4 (0.34 mg/mL) does not affect the lifespan of *Canton-S* males, *Canton-S* females, *w*^*1118*^ males, or *w*^*1118*^ females (S5C–S5F Fig), suggesting BH4 is nontoxic.

For further evidence the regulation of feeding by *pr* depends on its role in the production of BH4, we next asked whether loss-of-function of the other genes involved in BH4 synthesis replicates the *pr* loss-of-function phenotype. Indeed, we found fat body-specific knock-down of *Pu* using two different Pu-IR lines induces hyperphagia (Fig 3D and S7A Fig). Like that caused by the knock-down of *pr*, pre-feeding with low-dose BH4 rescues the hyperphagia caused by loss of *Pu* in the fat body (Fig 3D). We also confirmed that, as with *pr*, fat body-specific loss of *Pu* significantly enhances starvation resistance (S5C Fig).

Sptr catalyzes the final step in the biosynthesis of BH4. Thus, we next measured the appetite of the hypomorphic *Sptr*^*f04272*^ mutant strain. We found homozygous *Sptr*^*f04272*^ mutants eat more than *w*^*1118*^ flies, but pre-feeding them with low dose BH4 rescues this increase in appetite (Fig 3E). As with the *pr*^*G3141*^ mutant, the hyperphagia of the *Sptr*^*f04272*^ mutant was unaffected by seven generations of back-crossing to an isogenic *w*^*1118*^ strain (S3 Fig). Next, we reasoned if Purple acts upstream of Sptr with regard to this feeding phenotype, overexpression of *pr* in the adult fat body should not rescue the hyperphagia of *Sptr* mutants. To confirm this hypothesis, we used the ubiquitous temperature-sensitive tub-GAL80^ts^ to limit the overexpression of *pr* to the adult fat body in both the *w*^*1118*^ and *Sptr*^*f04272*^ genetic backgrounds. At 18°C, while GAL80 is active and *pr* is not being overexpressed, we did not observe any difference between the experimental lines and their corresponding heterozygous controls (Fig 3F, above). In flies shifted to 30°C post-eclosion, however, overexpression of *pr* in the fat body reduces the feeding of *w*^*1118*^ flies but not *Sptr*^*f04272*^ flies (Fig 3F, below).

### Sptr is required in adult NPF neurons

**Fig 4:**
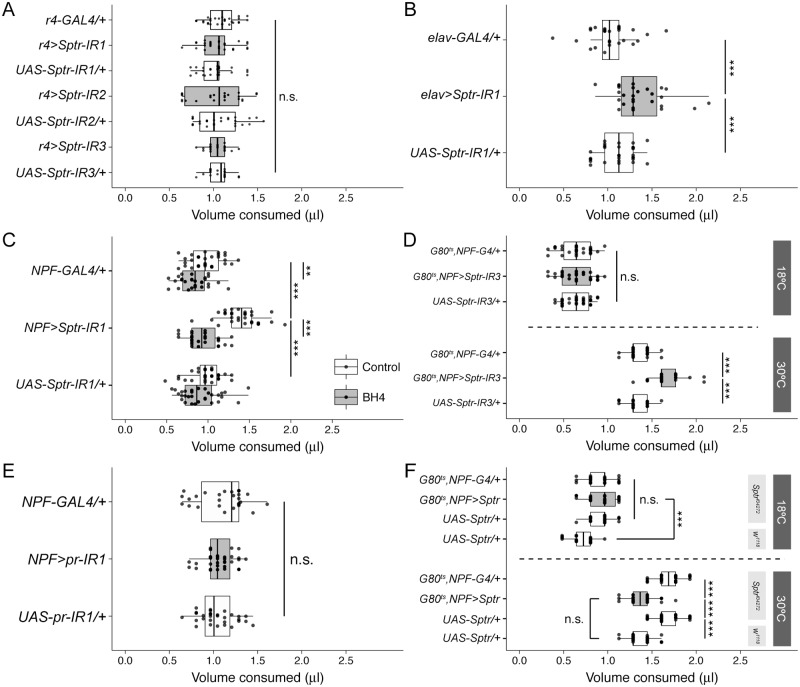
Proper appetite regulation depends on Sepiapterin Reductase (Sptr) expression in neuropeptide F (NPF) neurons, not the fat body. **(A)** Fat body-specific knock-down of *Sptr* using three different RNA intereference (RNAi) lines (gray boxplots) does not alter feeding compared to heterozygous controls (white boxplots; *n* = 20–29). See also S6A Fig. **(B)** Pan-neuronal knock-down of *Sptr* increases feeding (*n* = 27–28). See also S6C and S7 Figs. **(C)** Knock-down of *Sptr* in NPF neurons increases feeding, and this hyperphagia can be rescued by pre-feeding the flies 0.17 mg/mL tetrahydrobiopterin (BH4; *n* = 30). See also S8A and S8B Fig. **(D)** Limiting the NPF neuron-specific *Sptr* knock-down (gray boxplots) to the adult stage using a temperature-sensitive tub-GAL80^ts^ still increases feeding beyond that of heterozygous controls (white boxplots; *n* = 30). Importantly, there is no change in feeding at 18°C. **(E)** Knock-down of *pr* in NPF neurons has no effect on feeding amount (*n* = 27–34). **(F)** Adult stage NPF neuron-specific *Sptr* overexpression (gray boxplots) rescues the hyperphagia of *Sptr*^*f04272*^ mutant flies to the level of a *w*^*1118*^ background control (*n* = 30).

We next asked whether the role of *Sptr* in appetite regulation is, like that of *Pu* and *pr*, specific to the fat body. To our surprise, we were unable to observe any changes in feeding despite combining three different Sptr-IR lines with the fat body-specific drivers r4-GAL4 (Fig 4A) or FB-GAL4 (S7B Fig). Because feeding behavior is ultimately directed by the brain, we next used the pan-neuronal elav-GAL4 driver to knock-down *Sptr* in all post-mitotic neurons. We found that, indeed, neuron-specific knock-down of *Sptr* increases feeding (Fig 4B and S7C Fig). After confirming pan-neuronal knock-down of *pr* has no effect on feeding (S7D Fig), it became clear that the steps in the biosynthesis of the BH4 required for proper appetite control are physically distributed between the fat body and the brain. This suggests the intermediate metabolite PTP circulates, probably via the hemolymph, from its site of origin in the fat to its site of conversion to BH4 in the brain.

Next, we screened a group of neuronal GAL4 lines to more precisely define the *Sptr*-positive neurons that regulate feeding (S8 Fig). *Sptr* knock-down with both the ubiquitous Actin-5C driver (Act5C-GAL4) and the “pan-peptidergic” driver (386Y-GAL4) increase feeding (S8A and S8B Fig). Knock-down of *Sptr* using the broad dopaminergic (TH-GAL4) and serotonergic (Trh-GAL4) drivers as well as the “obesity blocking neuron” driver (c673a-GAL4) [24], the insulin-producing cell driver (dilp2-GAL4), the oenocyte driver (OK72-GAL4), and the feeding-related peptidergic neuron drivers sNPF-GAL4 and AstA-GAL4 do not affect feeding amount (S8C–S8I Fig). We did observe hyperphagia; however, when we combined a GAL4 driver that labels the small group of neurons that express the neuropeptide NPF with each of three different Sptr-IR lines (Fig 4C and S9A and S9B Fig). As expected, pre-feeding these flies with a low dose of BH4 also rescues this hyperphagia (Fig 4C and S9A Fig). To rule out the possibility this phenotype is a developmental artifact, we limited the knock-down of *Sptr* to adult NPF neurons by adding the ubiquitously expressed temperature-sensitive tub-GAL80^ts^. As expected, flies at 18°C eat similar amounts (Fig 4D, above), but when shifted to 30°C, flies with Sptr-depleted NPF neurons eat more than the corresponding heterozygous controls (Fig 4D, below). In support of the physical distribution of BH4 synthesis as it relates to appetite control, neither the NPF neuron-specific knock-down of *pr* (Fig 4E) nor *Pu* (S9C Fig) alters feeding amount. We also found that, as with *Pu* and *pr* in the fat body, NPF neuron-specific knock-down of *Sptr* enhances starvation resistance (S5D Fig). This suggests the increase in appetite observed with *Sptr* loss-of-function is also associated with a larger supply of mobilizable energy.

We next asked whether adult NPF neuron-specific overexpression of *Sptr* rescues the hyperphagia of *Sptr* mutants. At 18°C, flies carrying the *Sptr*^*f04272*^ mutation eat more than flies lacking this mutation regardless of their remaining genotype (Fig 4F, above). Shifting these genotypes to 30°C post-eclosion causes the *Sptr*^*f04272*^ mutant flies overexpressing *Sptr* in their NPF neurons to eat the same as the controls in the *w*^*1118*^ background and much less than their corresponding heterozygous controls in the *Sptr*^*f04272*^ background (Fig 4F, below). Similar overexpression of *Sptr* in the NPF neurons of flies in the *w*^*1118*^ background also reduces feeding (S9D Fig).

Thus, the production of BH4 necessary for feeding does seem to be physically distributed between the fat body and the NPF neurons. This conclusion is contingent, however, on r4-GAL4 and NPF-GAL4 having non-overlapping patterns of expression. As expected, when we used r4-GAL4 and NPF-GAL4 to drive expression of green fluorescence protein (GFP) and stained the fat body and brain of those flies with an anti-GFP antibody, we found no overlap in their expression patterns (S10 Fig). Underlying numerical data for this figure can be found here: http://dx.doi.org/10.5061/dryad.8hm82.

### BH4 induces satiety by inhibiting the NPF neurons

**Fig 5:**
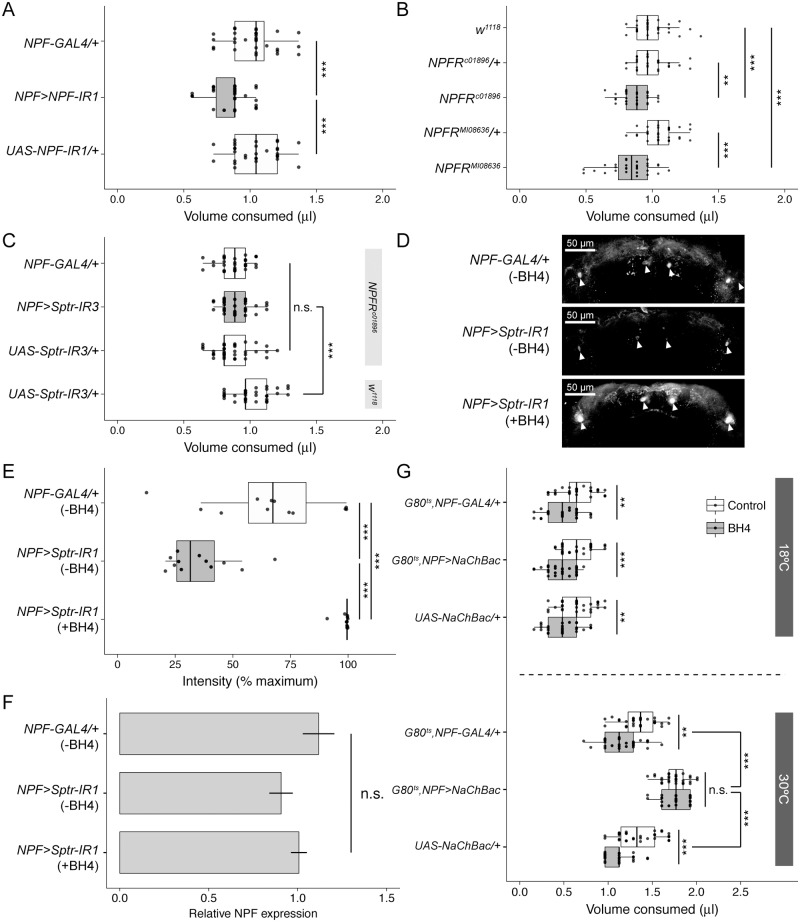
Tetrahydrobiopterin (BH4) acts via neuropeptide F (NPF)-NPFR signaling to modulate appetite. **(A)** NPF knock-down in NPF neurons (gray boxplot) reduces ad libitum feeding (*n* = 30) below that of heterozygous controls (white boxplots). **(B)** Two hypomorphic NPF receptor mutants, *NPFR*^*c01896*^ and *NPFR*^*MI08636*^ (gray boxplots), eat less than *w*^*1118*^ and their heterozygous controls (white boxplots; *n* = 30). **(C)** In the *NPFR*^*c01896*^ mutant background, knock-down of *Sepiapterin reductase* (*Sptr*) in NPF neurons fails to enhance feeding (*n* = 35). **(D)** Staining of dissected brains with an NPF-specific antibody. NPF neuron-specific knock-down of *Sptr* reduces NPF accumulation in NPF neuron cell bodies. Pre-feeding with 0.34 mg/mL BH4 dramatically increases NPF accumulation. Arrowheads indicate the NPF neuron cell bodies. **(E)** Boxplots comparing the NPF signal intensities of 12 stained cell bodies from three brains for each genotype. All brains were imaged with the same confocal settings. **(F)** Relative *NPF* gene expression normalized to three control genes under the same conditions as **(D)** and **(E)** as measured by quantitative polymerase chain reaction (qPCR). Each bar indicates the mean ± standard error of the mean (s.e.m.) of three technical replicates of each of two biological samples (RNA extracted from 40 adult brains) amplified using two different *NPF*-specific primer sets. **(G)** Continuous hyper-activation of adult NPF neurons using the bacterial sodium channel NaChBac induces hyperphagia. This hyperphagia is not rescued by pre-feeding with 0.34 mg/mL BH4 (*n* = 30). Underlying numerical data for this figure can be found here: http://dx.doi.org/10.5061/dryad.8hm82.

Next, we explored the mechanism by which BH4 functions in the NPF neurons to regulate feeding. NPF is the closest fly homologue of the mammalian appetite regulator NPY [25]. Although NPF plays a clear role in extending the foraging phase of larval development [26], the extent to which NPF loss-of-function affects ad libitum feeding in adult flies remains unclear. As we expected, though, knock-down of NPF in the NPF neurons reduces adult ad libitum feeding (Fig 5A). Consistent with this result, two different homozygous NPF receptor mutants, *NPFR*^*c01896*^ and *NPFR*^*MI08636*^, eat less than *w*^*1118*^ and their heterozygous controls (Fig 5B). We observed a similar result with *NPFR*^*c01896*^ after seven generations of back-crossing to an isogenic *w*^*1118*^ strain (S3 Fig). If the role of BH4 in the regulation of feeding acts via NPF-neuropeptide F receptor (NPFR) signaling, NPFR loss-of-function should mask the increase in feeding caused by NPF neuron-specific loss of *Sptr*. Indeed, in the *NPFR*^*c01896*^ mutant background, flies whose NPF neurons express the Sptr-IR3 transgene eat the same as heterozygous controls and less than a *w*^*1118*^ genetic background control (Fig 5C). Here, we only compared their feeding to the UAS control and not the GAL4 control in the *w*^*1118*^ background because both eat similar amounts (S8B Fig). We chose to use Sptr-IR3 instead of the other equally functional Sptr-IR lines because its genomic location made it easier to combine with the *NPFR*^*c01896*^ mutant background.

BH4 is a required cofactor of Nitric oxide synthase (Nos) and of the aromatic amino acid hydroxylases required to produce dopamine and serotonin [22]. These well-established relationships suggested a possible mechanism for the role of BH4 in feeding regulation. Contrary to our expectations, however, NPF neuron-specific knock-down of each of these enzymes—Nos, Tryptophan hydroxylase (Trh), Tyrosine hydroxylase (pale), and Phenylalanine hydroxylase (Henna)—using two different IR lines for each causes no change in feeding behavior (S11 Fig).

Because we confirmed the effect of BH4 on feeding acts via NPF-NPFR signaling, we next visualized the effects of BH4 manipulation on NPF levels in the adult brain using an NPF-specific antibody. We found that, when compared to a heterozygous control (Fig 5D and 5E, above), NPF neuron-specific knock-down of *Sptr* is associated with reduced staining of NPF in the brain (Fig 5D and 5E, middle). Pre-feeding these same flies with high-dose BH4 (0.34 mg/mL) dramatically increases NPF staining in their *Sptr*-depleted NPF neurons (Fig 5D and 5E, below). When considering only these NPF staining results, it seems possible that the loss of BH4 associated with *Sptr*-depletion somehow reduces NPF expression and BH4 treatment somehow induces NPF expression. To investigate this possibility, we extracted total RNA from the brains of these same three groups of flies and used it to measure NPF expression via quantitative polymerase chain reaction (qPCR). Although there does seem to be a slight reduction in NPF expression induced by *Sptr*-depletion, it is not statistically significant. In addition, pre-feeding the *Sptr*-depleted flies with BH4 does not alter NPF expression (Fig 5F). Because the changes in NPF staining cannot be attributed to changes in NPF gene expression, it suggests BH4 may instead be inhibiting NPF release—either directly or by strongly inhibiting the firing of the NPF neurons.

To investigate these two possibilities, we artificially activated the NPF neurons using the bacterial sodium channel NaChBac. To rule out any developmental artifacts caused by constitutive NPF neuron activation, we added the temperature-sensitive tub-GAL80^ts^. At 18°C, all genotypes eat similar amounts, more without and less with high dose BH4 pre-feeding (Fig 5G, above). At 30°C, flies with hyper-activated NPF neurons eat much more than the appropriate heterozygous controls regardless of BH4 pre-feeding (Fig 5G, below). The presence of NaChBac overcomes the effect of high-dose BH4 pre-feeding, suggesting the NPF neurons are still releasing NPF.

In addition to artificially activating the firing of the NPF neurons, we also blocked their release of NPF by driving their expression of the temperature-sensitive dynamin mutant Shibire^ts^ (Shi^ts^). We decided to do this experiment in both the *w*^*1118*^ and *pr*^*G3141*^ genetic backgrounds to once again verify that Purple functions upstream of NPF in appetite regulation. As with the artificial NPF neuron activation experiment, we included the temperature-sensitive tub-GAL80^ts^ to rule out any developmental effects caused by continuous blockade of NPF release. At the permissive temperature (18°C), vesicle release occurs normally, and flies with the *pr*^*G3141*^ genetic background eat more than *w*^*1118*^ (S12A Fig, above). At the restrictive temperature (30°C), NPF release is inhibited and flies with both the *w*^*1118*^ and *pr*^*G3141*^ genetic backgrounds eat less than the heterozygous controls (S12A Fig, below). We also found that inhibition of NPF release via expression of Shi^ts^ causes NPF to accumulate in the cell bodies of the NPF neurons (S12B and S12C Fig). Together, the reduction of NPF staining upon *Sptr*-depletion, the increased NPF staining upon BH4 treatment, and inhibition of NPF release strongly suggest BH4 is inhibiting the firing of the NPF neurons. Further experiments will be necessary to determine whether BH4 inhibits the NPF neurons directly by hyper-polarizing their membranes or indirectly via a negative feedback circuit.

### Dietary nutrient density affects the expression of BH4-related enzymes

**Fig 6:**
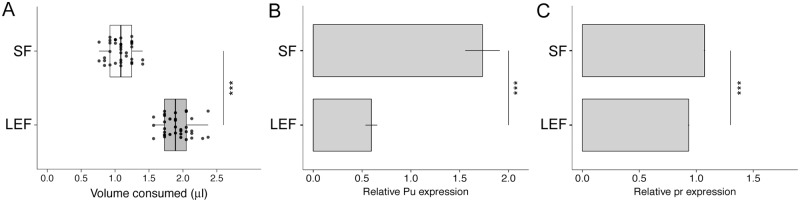
Reducing dietary nutrient density increases feeding and reduces the expression of tetrahydrobiopterin (BH4)-related enzymes. **(A)** Wild-type *Canton-S* flies maintained for 4 d on standard food (SF, *n* = 35) versus low-energy food (LEF; 1% sucrose solution, *n* = 35). LEF treatment significantly increases feeding in the capillary feeder (CAFE) assay. **(B)** Relative *Pu* gene expression normalized to three control genes under the same conditions as **(A)** as measured by quantitative polymerase chain reaction (qPCR). Each bar represents the mean ± standard error of the mean (s.e.m.) for three technical replicates of each of two biological samples (RNA extracted from 25 adult fly abdomens) amplified using two different *Pu*-specific primer sets. **(C)** Relative *pr* gene expression normalized to three control genes under the same conditions as **(A)** as measured by qPCR. Each bar represents the mean ± s.e.m. for three technical replicates of each of three biological samples (RNA extracted from 25 adult fly abdomens) amplified using two different *pr*-specific primer sets. Underlying numerical data for this figure can be found here: http://dx.doi.org/10.5061/dryad.8hm82.

Finally, to further assess the physiological relevance of our findings, we asked whether dietary manipulation alters the expression of BH4-related enzymes in wild-type flies. As the catalyst for the first and rate-limiting step in the metabolic pathway that leads to BH4 synthesis, Punch is likely subject to the most transcriptional regulation. We observed loss-of-function for all the BH4-related enzymes increase appetite while their gain-of-function reduces appetite. Thus, we expected a dietary manipulation that increases feeding to reduce Punch expression. To manipulate feeding, we subjected adult wild-type (*Canton-S*) flies to either 4 d of standard food (SF) or 4 d of low-energy food (LEF) comprising only a 1% sucrose solution. We moved these flies to our modified CAFE assay, acclimated them for 24 h, and measured the feeding of each group on a 5% sucrose solution for 32 h. As expected, LEF flies food eat significantly more than SF flies (Fig 6A). We next isolated total RNA from abdomens (containing the fat body) dissected from each group of flies. Then, we prepared cDNAs and measured the expression of *Pu* and *pr* using qPCR. Not only does LEF treatment significantly reduce *Pu* expression (Fig 6B) but it also reduces *pr* expression (Fig 6C). This result is consistent with the role we have identified for BH4-related enzymes in the regulation of feeding.

## Discussion

**Fig 7:**
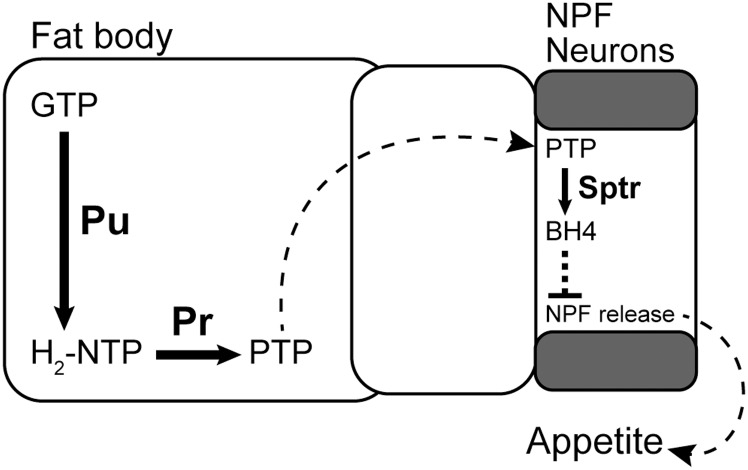
A model for the function of tetrahydrobiopterin (BH4) in the regulation of appetite. Punch and Purple are required in the fat body to generate 6-pyruvoyltetrahydropterin (PTP) from guanosine triphosphate (GTP). In our model, PTP is then transferred to the brain where it is converted to BH4 in the neuropeptide F (NPF) neurons. Through an as yet unknown mechanism, BH4 seems to reduce NPF release, in turn, reducing feeding. Solid arrows indicate single-step enzymatic conversions and dotted lines indicate what are likely multi-step processes.

This study began with a genetic screen for fat-derived modulators of feeding. In this screen and the experiments that followed, we identified a new function for the enzymatic cofactor BH4 and thus the enzymes required to produce it (i.e., Punch, Purple, and Sptr) in reducing appetite in adult *Drosophila*. We found while appetite regulation requires Punch and Purple expression in the fat body, it requires Sptr expression in the brain. We also found pre-feeding flies BH4 rescues the increases in feeding caused by loss-of-function of Punch and Purple in the fat body and Sptr in the brain. Thus, the steps in the biosynthesis of the BH4 required for appetite regulation are physically distributed between the fat body and the brain, implying the circulation of a fat body-derived PTP intermediate (Fig 7).

Despite Punch and Purple each catalyzing reactions that create small molecule intermediates that could readily circulate through the hemolymph of the open insect circulatory system, there seems to be a strong tissue specificity to their expression patterns. The developing eyes are the sites of highest expression of both Punch and Purple where they function in drosopterin eye pigment production rather than BH4 production. The *Pu* locus produces at least three transcripts that each show temporal and tissue-specific expression patterns as well as differential regulation by BH4 [27]. The *pr* locus produces two transcripts [20]—one is eye-specific for pigment production and the other is more broadly expressed (e.g., in the fat body) for BH4 synthesis [21]. Again, our results suggest that PTP produced in the fat body by the successive action of Punch and Purple circulates for conversion to BH4 by Sptr in the NPF neurons. Our results also imply the PTP produced by Punch and Purple in the eye does not circulate for conversion to BH4 by Sptr in the NPF neurons. This could be because the PTP to drosopterin conversion occurs too fast or because the fat body expresses an unknown PTP transporter absent in the eye. Regardless, the fat body-specific expression of Purple and Punch necessary for appetite regulation makes it tempting to speculate that fat-derived PTP may signal an energy replete status to the brain reducing the drive to eat. We have already shown reducing dietary nutrient content enhances feeding and reduces the expression of Punch and Purple, but it will also be interesting in the future to see whether the expression or activity of either Punch or Purple in the fat body changes with fat storage volume or lipolysis.

Considering the feeding phenotypes caused by fat body-specific knock-down of *Pu* and *pr*, the lack of any phenotype caused by fat body-specific knock-down of *Sptr* surprised us. Still, after a small, targeted screen of neuronal GAL4 lines we were able to narrow the necessary feeding-related expression of Sptr to two pairs of bilaterally symmetric dorsolateral brain neurons expressing the neuropeptide NPF. Because FlyAtlas data reveal “moderate” expression of Sptr in the brain, these four NPF neurons are almost certainly not the only Sptr-positive neurons. It will be interesting to identify other Sptr-positive neurons and determine whether they also contribute to appetite regulation or other behavioral phenotypes. If other Sptr-positive neurons do exist, the inability of the Sptr they produce to compensate for the knock-down of *Sptr* in the NPF neurons suggests the locations that require BH4 must produce it directly themselves. This is either because BH4 itself does not circulate at all or because it is rapidly degraded when it does. We did observe successful rescues with BH4 pre-feeding, but the rescue doses were likely much larger than that produced endogenously for immediate and local use. The BH4 pre-feeding rescues also suggest only that exogenous BH4 can enter the cells that need it, not that it can exit. We speculate that the physical distribution of BH4 synthesis we have explored here may provide flies with a way to regulate BH4 levels on both the global and local scales. Global regulation of BH4 levels could be accomplished by altering the expression of *Pu* or *pr* in the fat body, while local regulation of BH4 levels—even at the level of an individual neuron—could be accomplished by regulating *Sptr* expression.

Once we implicated the NPF neurons, we began to investigate possible mechanisms underlying their role in the BH4-dependent regulation of feeding. Using a combination of genetic techniques aimed at altering the firing rate and function of the NPF neurons, we found that BH4 seems to inhibit feeding by inhibiting the NPF neurons and their release of NPF. The mechanism by which BH4 does this, though, is still unclear. Although the aromatic amino acid hydroxylases as well as Nos are known to require BH4 as a cofactor, we were unable to observe any changes in feeding induced by NPF neuron-specific knock-down of any of these enzymes (S11 Fig). It is possible that BH4 acts in the NPF neurons as a cofactor of some unknown enzyme. It is also possible, although unlikely considering the strength of the NPF-GAL4 line, that the knock-down of the known BH4-dependent enzymes was insufficient. In the future, we hope to clarify this mechanism by which BH4 inhibits the NPF neurons.

The enzymes that act in the BH4 biosynthetic pathway are all well-studied, at least in part because their mutations lead to phenylketonuria or hyperphenylalanemia in humans. The severe developmental abnormalities that characterize these diseases stem mainly from a toxic build-up of phenylalanine and an absence of the neurotransmitters (e.g., dopamine and serotonin) derived from aromatic amino acids [22]. Murine models of BH4 deficiencies also show severe developmental defects. PTPS (Purple) mutant mouse pups die within 48 h after birth [28], and the loss of dopamine in Sptr (SPR in the murine model) mutant mice leads to severe dwarfism [29]. It may be puzzling that the BH4-deficiency phenotypes we observe in flies are so much milder than those observed in mice and humans, but it is important to note that the fly phenotypes we have observed are not caused by total loss-of-function. Using the temperature-sensitive tub-GAL80^ts^, we were able to temporally limit many of our knock-down experiments to the adult stage. Because the enzymes that produce BH4 and the enzymes that require BH4 are all highly conserved, it will be interesting to see whether our results in flies also apply to mice or humans. It is possible, for example, mutations affecting individual enhancers of a gene like human SPR (Sptr) could reduce its expression in limited populations of neurons, causing subtle behavioral phenotypes. This would mean the severity of the known BH4-deficient syndromes is masking much less severe but important phenotypes. Perhaps further experiments in flies will be useful in directing a more careful examination of mouse models of BH4-deficiency as well.

## Materials and Methods

### Fly stocks

We maintained our fly stocks at either 18°C or 25°C and 60% humidity under a 12h:12h light:dark (LD) cycle on standard cornmeal-yeast-corn syrup medium (http://flystocks.bio.indiana.edu/Fly_Work/media-recipes/bloomfood.htm). The only modification in our fly food recipe is the addition of 1.5 g Tegosept per liter of food as an antifungal agent.

We generated the UAS-miRNA stock library as previously described [12]. To generate *UAS-pr* and *UAS-Sptr*, we amplified appropriate ORFs via PCR and inserted them into the pUASTattB vector [30]. Then, using standard methods, we micro-injected the resulting constructs for site-specific insertion into embryos containing attP landing sites. The primers (5′ to 3′) we used were: UAS-Sptr (CAATCATGGACCTGAAACA and CTAGAACTGCTCATCCCT); UAS-pr (TTGAAATGTCGCAGCAA and TGAGCGATGCGTTTG). We injected *UAS-pr* into an attP site on the third chromosome (Bloomington Stock # 9750) and *UAS-Sptr* into an attP site on the second chromosome (Bloomington Stock # 9752).

The following flies are described elsewhere: S_1_106-GAL4 [13], FB-GAL4 [18], TH-GAL4 [31], Trh-GAL4 [32], c673a-GAL4 [24], Dilp2-GAL4 [33], sNPF-GAL4 [34], AstA-GAL4 [35], and UAS-Shibire^ts^ [36].

We obtained the following fly stocks (stock number) from the Bloomington Stock Center: Canton-S (1), w^1118^ (3605), isogenic w^1118^ (5905), pr^1^ (370), pr^G3141^ (27097), r4-GAL4 (33832), tubP-GAL80^ts^ (7018, 7019), Sptr^f04272^ (18753), elav-GAL4 (8760), 386Y-GAL4 (25410), GMR-GAL4 (1104), NPF-GAL4 (25681), Act5C-GAL4 (4414), OK72-GAL4 (6486), NPFR^MI08636^ (52133), NPFR^c01896^ (10747), UAS-NaChBac (9466), 10xUAS-myR::GFP (32198), UAS-Pu-IR1 (41998), UAS-pr-IR2 (53346), UAS-Sptr-IR1 (28968), UAS-Sptr-IR2 (43277), UAS-NPF-IR1 (27237), UAS-Nos-IR2 (28792), UAS-Trh-IR2 (25842), UAS-ple-IR2 (25796), UAS-Hn-IR2 (29540).

We obtained the following fly stocks (stock number) from the VDRC Stock Center: UAS-Pu-IR2 (v107296), UAS-pr-IR1 (v109430), UAS-Sptr-IR3 (v17018), UAS-Nos-IR1 (v108433), UAS-Trh-IR1 (v105414), UAS-ple-IR1 (v108879), UAS-Hn-IR1 (v110511).

We used the Updated Targets of RNAi Reagents (UP-TORR) search tool (http://www.flyrnai.org/up-torr/) to identify any predicted off-targets for all the RNAi lines we used. While most of the RNAi reagents we used have no predicted off-targets, for the few that do, we found no common predicted off-targets between the 2–3 RNAi reagents we tested for each target gene.

### Genetic back-crossing

To clarify the extent to which genetic background affects our primary results, we back-crossed the *pr*, *Sptr*, and *NPFR* mutant strains for seven generations to an isogenic *w*^*1118*^ strain. We then compared the feeding of the resulting back-crossed homozygous mutant strains to heterozygous strains (crossed again to *w*^*1118*^), to the isogenic *w*^*1118*^ strain, and to *Canton-S*.

### Feeding assays

To quantify ad libitum feeding in adult flies, we modified the CAFE assay described by Ja et al. [14]. Unless otherwise noted, we used 5-d-old males for all assays. For each assay, we transferred two flies to a standard fly vial containing only a wet Kimwipe. We then introduced a calibrated 20-μl capillary (Marienfeld Superior [2920110], Germany) filled with 5% sucrose in water between a sponge plug and the side of the vial. We allowed the flies to acclimate for 24 h before refilling the capillary. Finally, we allowed the flies to feed for 32 h without replacing the capillaries before measuring total consumption. We controlled for evaporation by subtracting the volume lost from capillaries in identical vials without flies. As environmental humidity and evaporation rates varied over time, we always tested the experimental and control genotypes in each experiment simultaneously to maximize the reliability of our results. When we report *n* in the figure legends, it refers to the number of CAFE assays, with each representing the collective feeding of two flies.

For all experiments using the S_1_106-GAL4 line, we transferred 4-d-old males to the vial containing a wet Kimwipe and starved them for 24 h. We then introduced a capillary containing 0.01 M RU486 (Sigma-Aldrich, South Korea) and 5% sucrose in water for 24 h. The subsequent CAFE assays were identical to those described above.

For temperature-sensitive tub-GAL80^ts^ experiments, we reared the flies at 18°C until eclosion. We then left half the flies in 18°C as negative controls, performing the CAFE assays as described above at 18°C. We shifted the remaining flies to 30°C immediately after eclosion and performed the CAFE assays as described above.

For experiments involving BH4 treatment, we included either 0.17 or 0.34 mg/mL BH4 (Schircks Laboratories [11.212–5], Switzerland) in the 5% sucrose solution provided during the 24 h acclimation stage. Then, we replaced the capillary with one containing only 5% sucrose for the feeding measurement. For the immunostaining and qPCR experiments, we fed 5-d-old flies for 2 d from capillaries containing 0.34 mg/mL BH4 in 5% sucrose. To avoid contamination and any chance that it would lose its effectiveness, we prepared fresh BH4-sucrose solutions every day.

For the short-term CAFE assay, we modified the standard CAFE assay described above to track feeding over the course of 24 h with minimal disruption to the flies. We accomplished this by adding a blue dye (FD&C Blue No. 1, 0.2 mg/mL) to the 5% sucrose solution and taking pictures of the assay vials every 4 h. We then calculated the volume changes using the image analysis software ImageJ [37]. The flies treated with BH4 received the “low” dose of 0.17 mg/mL added directly to the sucrose solution. We also used five male flies per vial instead of two.

For the *NPF>Shi*^*ts*^ experiment, we reared the flies at 18°C. After one day of habituation in the CAFE assay at 18°C, we raised the temperature to 30°C to block synaptic transmission in the NPF neurons. After 24 h at 30°C, we started a standard CAFE assay and then dissected the flies for brain staining of NPF.

We performed additional feeding assays using ^32^P-labeled food as previously described [19]. Briefly, we habituated adult flies on normal food after eclosion. On day 5, we transferred groups of 10 male flies to vials containing food with 0.4 nCi/μL [α-^32^P] dCTP (PerkinElmer). After 24 h, we transferred the flies to empty vials and allowed them to groom for 15 min. We then killed the flies by freezing and move them to 4 mL of scintillation fluid (Ultima Gold, PerkinElmer) for counting in a multipurpose scintillation counter (Tri-Carb 2910 TR, PerkinElmer). We subtracted the scintillation counts for control flies fed non-labeled food from the experimental readings and estimated the volume of food consumed using the reading from a 100 μL aliquot of radiolabeled food.

### Immunostaining

We carried out whole-mount brain immunofluorescence as previously described [38] using an anti-NPF primary antibody at 0.05 μg/μL (RayBiotech [RB-19-001], US) and a goat anti-rabbit secondary antibody coupled to Alexa Fluor 488 (1:1000, Life technologies, US). We imaged the brains with a 20X objective on an LSM780 confocal microscope (Zeiss, Germany) and quantified staining intensities of individual cells using ImageJ [37].

To verify GAL4 line expression patterns, we crossed NPF-GAL4 and r4-GAL4 to UAS-GFP and then dissected the fat body, brain, and ventral nerve cord from 5-d-old male flies. After fixing these tissues with 4% paraformaldehyde in PBS, we stained them with a rabbit anti-GFP antibody (1:1000, A11122, Invitrogen, US) and a goat anti-rabbit secondary antibody coupled to Alexa Fluor 488 (1:1000, Life technologies, US).

### Quantitative RT-PCR

After extracting total RNA from 40 adult brains per sample (*NPF*) or 25 adult abdomens per sample (*Pu* and *pr*) using Trizol (Invitrogen, US), we synthesized cDNAs using the SuperScript III First-Strand Synthesis System (Invitrogen, US). We then performed qPCR amplification using the TOPreal qPCR 2X PreMIX (Enzynomics, South Korea) and a CFX96 Real Time System (Bio-Rad, US). We used *rp49*, *actin*, and *α-tubulin* [39] for normalization and quantified relative expression using the Bio-Rad CFX Manager 3.1 software package. The primers (5′ to 3′) were as follows: *NPF* 1 (GATGCCTACAAGTTCCTGCAGG and GTGACGTTGCCATGGTCGT); *NPF* 2 (GCTCGCGGTTTTAATGAGGAGG and CTGTAGCTGCAACTACTTGTTCTTTCC); *Pu* 1 (ACTAAGCAGATCGCAGTGGC and CCTCACGGGTCTTGGGATC); *Pu* 2 (GAAATATTTTCGCGTCGCCTGC and CGTCCTCTAGTGCCCACTCTA); *pr* 1 (CACCGAGCTGAAAGAGGCT and GCGATGCGTTTGTTGATGGG); *pr* 2 (GAGATAACCGTCCGTGGTCC and TCTTCAGCTGCAGACGGATG); *rp49* (ATGACCATCCGCCCAGCATA and GAGAACGCAGGCGACCGTTG); *actin* (GCGTCGGTCAATTCAATCTT and AAGCTGCAACCTCTTCGTCA); *α-tubulin* (TGTCGCGTGTGAAACACTTC and AGCAGGCGTTTCCAATCTG).

### Lifespan measurements

We assessed the long-term toxicity of BH4 treatment using lifespan measurements. Briefly, we placed 100 flies in groups of 20 into vials containing either standard cornmeal food with or without BH4 at a final concentration of 0.34 mg/mL. We then counted the number of dead flies while transferring each group to new vials every 2 d.

### LEF

We used a 1% sucrose solution for the LEF condition. We separated male *Canton-S* flies 1 d post-eclosion into vials containing either SF or LEF and allowed them to feed for 4 d. Then, we divided these flies into two groups: one for the CAFE assay and the other for RNA isolation and qPCR.

### TAG assay

To measure TAGs, we homogenized ten 15-d-old adult male flies for each genotype and followed a standard protocol [40] using a DU 730 Spectrophotometer (Beckman Coulter, US). We calculated TAG levels by subtracting the amount of free glycerol in a PBS-treated sample from the total glycerol present in a similar sample treated with the triglyceride reagent (Serum Triglyceride Determination Kit; Sigma TR0100). We normalized the TAG levels to the protein level in each homogenate as determined by the Bradford assay (Bio-Rad).

### Activity measurement

We measured locomotor activity as previously described [41]. We collected adult males at eclosion and aged them in groups of 10–15 for 3 d. We loaded individual 3-d-old flies in 65 x 5 mm glass tubes containing 4% sucrose and 2% agar and measured their locomotor activity in 1 min bins with a DAM System monitor (Trikinetics) inside an incubator at 25°C and 50% humidity. We monitored the flies for 4 d under a 12:12 h LD cycle and compute their activity parameters from days 3 and 4 using custom software. The software designated uninterrupted periods of inactivity lasting at least 5 min as sleep bouts and excluded flies with no activity on the final day from the analysis. Total activity is the average number of daily beam crossings and waking activity is the beam crossings per minute while flies are awake.

### Body measurements

We measured body size as previously described [42]. To avoid overcrowding artifacts, we maintained 30 embryos per vial on standard cornmeal food. We measured body lengths of 1-d-old adult males from the anterior end of the head to the posterior end of the abdomen using ImageJ [37].

We measured dry weight as previously described [43]. Briefly, we dried groups of ten 1-d-old male flies for 24 h at 60°C. We then weighed them as a group and calculated the average dry weight per fly.

### Starvation resistance

We measured starvation resistance using the standard technique [44] with the following minor modification: each standard fly vial contained two adult male flies (6–8 d old) and a wet Kimwipe (Kimtech Science) to provide water.

### Statistics and reproducibility

We performed each experiment at least three times and found that all the results fell into similar ranges with no significant batch effects. Thus, the figures include all replications for each genotype. All boxplots in this study follow the standard Tukey style. We determined statistical significance by performing ANOVA analyses followed by pairwise *t* tests, correcting for multiple comparisons with the Bonferroni method. We designated all statistically significant results with asterisks (*** *p* ≤ 0.001, ** *p* ≤ 0.01, * *p* ≤ 0.05). In all figures, the *p* values of relevant statistical comparisons labeled with “n.s.” fell below these thresholds and were therefore non-significant. We did not label the significance of some comparisons, particularly those that were not biologically meaningful. We used the R programming language for all statistical analysis and for figure generation [45]. The following R packages were particularly valuable: dplyr [46], ggplot2 [47], plyr [48], survival [49], survminer [50], and tidyR [51].

Data deposited in the Dryad repository: http://dx.doi.org/10.5061/dryad.8hm82 [52].

## Supporting information

S1 Fig Summary of the primary miRNA over-expression screen. A ranked barplot showing the mean volume consumed per fly ± s.e.m. for the *S_1_106-GAL4/+* control (green), the *S_1_106>miRNA* experimental lines (red), and the *UAS-miRNA/+* controls (blue). Arrows indicate *S_1_106>miR-iab-4* and *UAS-miR-iab-4/+*. Underlying numerical data for this figure can be found here: http://dx.doi.org/10.5061/dryad.8hm82.
(TIF)

S2 Fig Feeding of female *pr* mutants. To determine whether *pr* also affects feeding in females, we measured *ad libitum* feeding in 5-day-old virgin females. Both *pr^G3141^* and *pr^1^* (gray) eat more than the *w^1118^* and *Canton-S* controls (white) (n = 21–22). Underlying numerical data for this figure can be found here: http://dx.doi.org/10.5061/dryad.8hm82.
(TIF)

S3 Fig Feeding of isogenized *pr*, *NPFR*, and *Sptr* mutants. To assess the effect of genetic background on our feeding results, we back-crossed *pr^G3141^*, *Sptr^f04272^*, and *NPFR^c01896^* to an isogenized w1118 strain for seven generations and then measured their feeding behavior. The isogenized *pr^G3141^* and *Sptr^f04272^* mutants showed increased feeding, while the *NPFR^c01896^* mutant showed reduced feeding compared to the isogenic *w^1118^* strain and *Canton-S* (n = 30). Underlying numerical data for this figure can be found here: http://dx.doi.org/10.5061/dryad.8hm82.
(TIF)

S4 Fig Physiological effects of fat body-specific *pr* knock-down. (A and B) Fat body-specific knock-down of *pr* does not significantly affect (A) body size (n = 81–117) or (B) dry weight (n = 10–15) as measured 1 day after eclosion. (C and D) Fat body-specific knock-down of *pr* does not significantly affect (C) total locomotor activity or (D) waking activity (n = 30–32). Underlying numerical data for this figure can be found here: http://dx.doi.org/10.5061/dryad.8hm82.
(TIF)

S5 Fig BH4-related genes and energy storage and metabolism. (A) Fat body-specific knock-down of *pr* induces a mild increase in whole body triacylglycerides (TAGs). TAG levels were normalized to protein levels (n = 5). (B) Fat body-specific knock-down of *pr* increases starvation resistance (n = 100). (C) Fat body-specific knock-down of *Pu* increases starvation resistance (n = 100). (D) NPF neuron-specific knock-down of *Sptr* increases starvation resistance (n = 100). For B–D, we made pair-wise comparisons of each heterozygous control line to the RNAi-expressing experimental line using the log-rank test. The reported *p*-value is the higher of these two values. Underlying numerical data for this figure can be found here: http://dx.doi.org/10.5061/dryad.8hm82.
(TIF)

S6 Fig BH4 feeding effect onset and long-term BH4 toxicity. (A and B) Modified short-term CAFE assay measuring cumulative consumption every 4 hours for 24 hours. The hypomorphic *pr* mutants (i.e., *pr^1^* and *pr^G3141^*) eat significantly more than wild-type controls (i.e., *w^1118^* and *Canton-S*) within 12 hr. Mixing a low dose of BH4 (0.17 mg/mL) into the feeding capillaries does not affect the consumption rates of the wild-type strains, but it rescues the increased feeding of the *pr* mutants within 16–20 hrs of feeding initiation (n = 27). (C–F) Continuous treatment with high dose BH4 (0.34 mg/mL) does not affect the lifespan of (C) *Canton-S* males, (D) *Canton-S* females, (E) *w^1118^* males, or (F) *w^1118^* females, suggesting BH4 is non-toxic. Underlying numerical data for this figure can be found here: http://dx.doi.org/10.5061/dryad.8hm82.
(TIF)

S7 Fig With regard to its role in feeding, BH4 synthesis is split between the fat body and the brain. (A) Fat body-specific knock-down of *Pu* using a second RNAi line (UAS-Pu-IR2) increases feeding (n = 25). (B) Knock-down of *Sptr* using a second fat body GAL4 line (FB-Gal4) does not affect feeding (n = 22–23). (C) Pan-neuronal knock-down of *Sptr* using a second RNAi line (UAS-Sptr-IR3) increases feeding (n = 23). (D) Pan-neuronal knock-down of *pr* does not affect feeding (n = 25–26). Underlying numerical data for this figure can be found here: http://dx.doi.org/10.5061/dryad.8hm82.
(TIF)

S8 Fig Directed screen to localize the role of Sptr in feeding regulation. (A) Ubiquitous knock-down of *Sptr* using Act5c-GAL4 increases feeding (n = 21). (B) Knock-down of *Sptr* in most peptidergic neurons using the enhancer trap 386Y-GAL4 increases feeding (n = 23–25). (C) Knock-down of *Sptr* in dopaminergic neurons using TH-GAL4 does not affect feeding (n = 23–25). (D) Knock-down of *Sptr* in serotonergic neurons using Trh-GAL4 does not affect feeding (n = 30). (E) Knock-down of *Sptr* in “obesity blocking neurons” using the enhancer trap c673-GAL4 does not affect feeding (n = 25). (F) Knock-down of *Sptr* in the insulin producing cells using dilp2-GAL4 does not affect feeding (n = 22–23). (G) Knock-down of *Sptr* in oenocytes using OK72-GAL4 does not affect feeding (n = 18–19). (H) Knock-down of *Sptr* in short neuropeptide F neurons using sNPF-GAL4 does not affect feeding (n = 18–19). (I) Knock-down of *Sptr* in allatostatin-A neurons using AstA-GAL4 does not affect feeding (n = 24–25). Underlying numerical data for this figure can be found here: http://dx.doi.org/10.5061/dryad.8hm82.
(TIF)

S9 Fig Manipulation of BH4-related genes in NPF neurons. (A) NPF neuron-specific knock-down of *Sptr* using a second RNAi line (UAS-Sptr-IR2) induces a hyperphagic phenotype that is rescued by pre-feeding the flies with 0.17 mg/ml BH4 (n = 24–30). (B) NPF neuron-specific knock-down of *Sptr* with a third RNAi line (UAS-Sptr-IR3) increases feeding (n = 26–27). (C) NPF neuron-specific knock-down of *Pu* does not affect feeding (n = 20). (D) NPF neuron-specific over-expression of *Sptr* reduces feeding (n = 40). Underlying numerical data for this figure can be found here: http://dx.doi.org/10.5061/dryad.8hm82.
(TIF)

S10 Fig Verification of the r4-GAL4 and NPF-GAL4 expression patterns in the fat body and CNS. (A) Staining of the abdominal fat body with a GFP-specific antibody shows that r4-GAL4 but not NPF-GAL4 drives expression in the fat body. Dotted lines mark the edges of the abdomen. Scale bars = 100 μm. (B) Staining of the adult brain and ventral nerve cord with a GFP-specific antibody shows that NPF-GAL4 but not r4-GAL4 drives expression in the brain. Scale bars = 100 μm.
(TIF)

S11 Fig NPF neuron-specific knock-down of enzymes known to require BH4 does not affect feeding. (A and B) Nitric oxide synthase (A, n = 30; B, n = 27–35). (C and D) Tryptophan hydroxylase (C, n = 28; D, n = 30). (E and F), pale (Tyrosine hydroxylase) (E, n = 20; F, n = 30). (G and H), Henna (Phenylalanine hydroxylase) (G, n = 22; H, n = 40). Underlying numerical data for this figure can be found here: http://dx.doi.org/10.5061/dryad.8hm82
(TIF)

S12 Fig Conditional inhibition NPF release. (A) Conditional inhibition of the NPF neurons with temperature-sensitive Shibire (Shi^ts^). At the permissive temperature (18°C), flies with the *pr^G3141^* mutant background eat more than those with the *w^1118^* background (n = 25). Inhibition of the NPF neurons at 30°C reduces the feeding of flies with both the *pr^G3141^* and *w^1118^* backgrounds (n = 25), confirming Purple functions upstream of the NPF neurons. (B) Staining of dissected brains with an NPF-specific antibody. Inhibition of the NPF neurons at 30°C (center) increases NPF accumulation in the NPF neuron cell bodies compared to controls. Arrowheads indicate NPF neuron cell bodies. All brains were imaged with the same confocal settings. (C) Boxplots comparing the NPF signal intensities of 12 stained cell bodies from 3 brains for each condition. Inhibition of the NPF neurons presumably increases the accumulation of NPF by blocking its release. Underlying numerical data for this figure can be found here: http://dx.doi.org/10.5061/dryad.8hm82
(TIF)

S1 Table miRNA Screen Results. (XLS)

S2 Table RNAi Screen Results. (XLS)

## Abbreviations

BH4: tetrahydrobiopterin
CAFE: capillary feeder
GFP: green fluorescence protein
GTP: guanosine triphosphate
H_2_-NTP: 7,8-dihydroneopterin triphosphate
IR: inverted repeat
LEF: low-energy food
miRNA: microRNA
Nos: Nitric oxide synthase
NPF: neuropeptide F
NPFR: neuropeptide F receptor
NPY: neuropeptide Y
*pr*: *purple*
PTP: 6-pyruvoyltetrahydropterin
PTPS: 6-pyruvoyltetrahydropterin synthase
*Pu*: *Punch*
qPCR: quantitative polymerase chain reaction
RNAi: RNA interference
SF: standard food
Shi^ts^: Shibire^ts^
Sptr: Sepiapterin Reductase
TAG: triacylglyceride
Trh: Tryptophan hydroxylase
UAS: upstream activation sequence
Upd2: Unpaired-2
