# Supplementary figures and images for: A fat-derived metabolite regulates a peptidergic feeding circuit in Drosophila
Source: PLoS Biol. 2017 Mar 28;15(3):e2000532. doi: 10.1371/journal.pbio.2000532 (PMC5369665; doi:10.1371/journal.pbio.2000532)

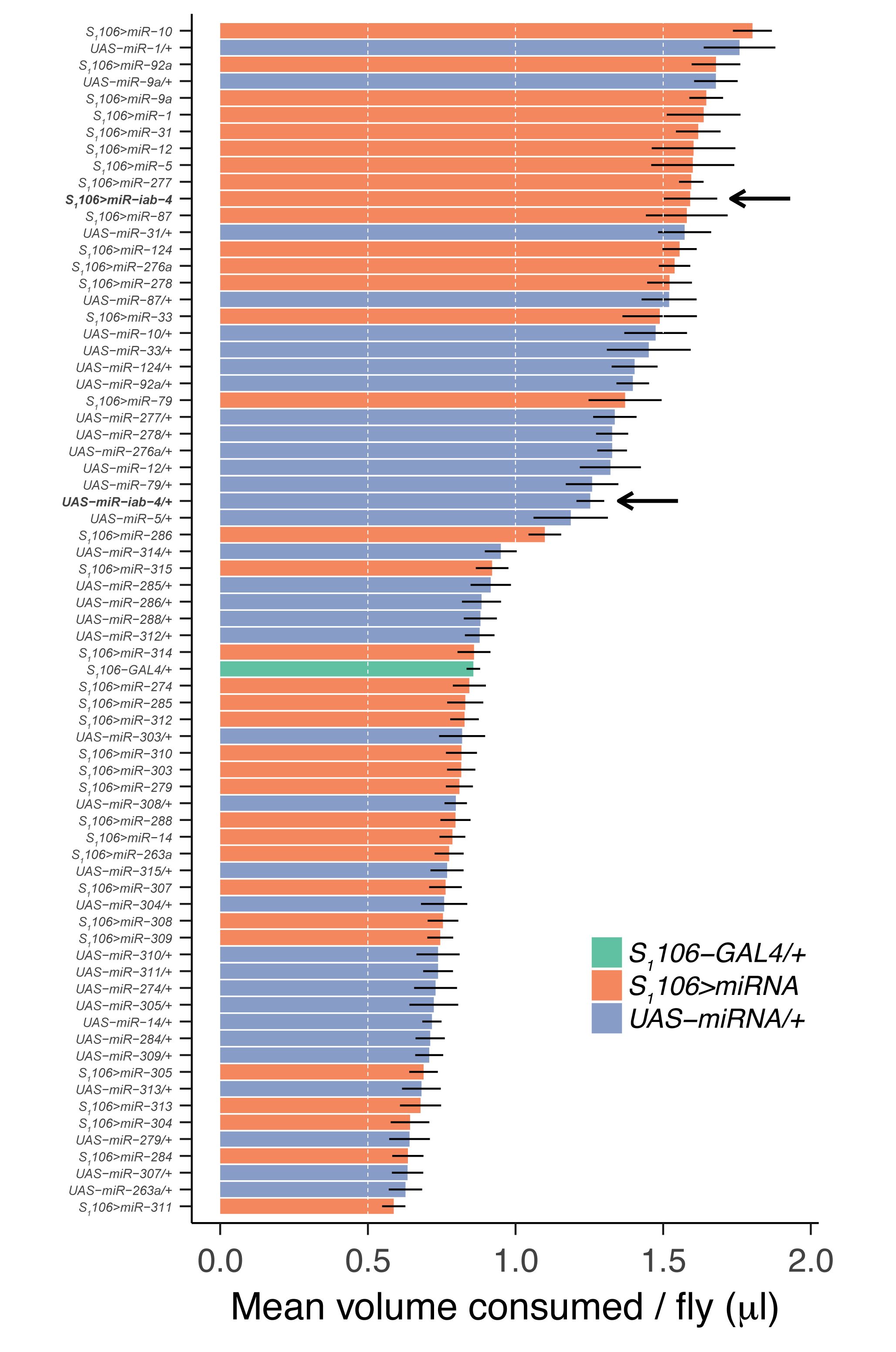

Supplement: S1 Fig — A ranked barplot showing the mean volume consumed per fly ± s.e.m. for the S1106-GAL4/+ control (green), the S1106>miRNA experimental lines (red), and the UAS-miRNA/+ controls (blue). Arrows indicate S1106>miR-iab-4 and UAS-miR-iab-4/+. Underlying numerical data for this figure can be found here: http://dx.doi.org/10.5061/dryad.8hm82. (TIF) [file pbio.2000532.s001.tif]

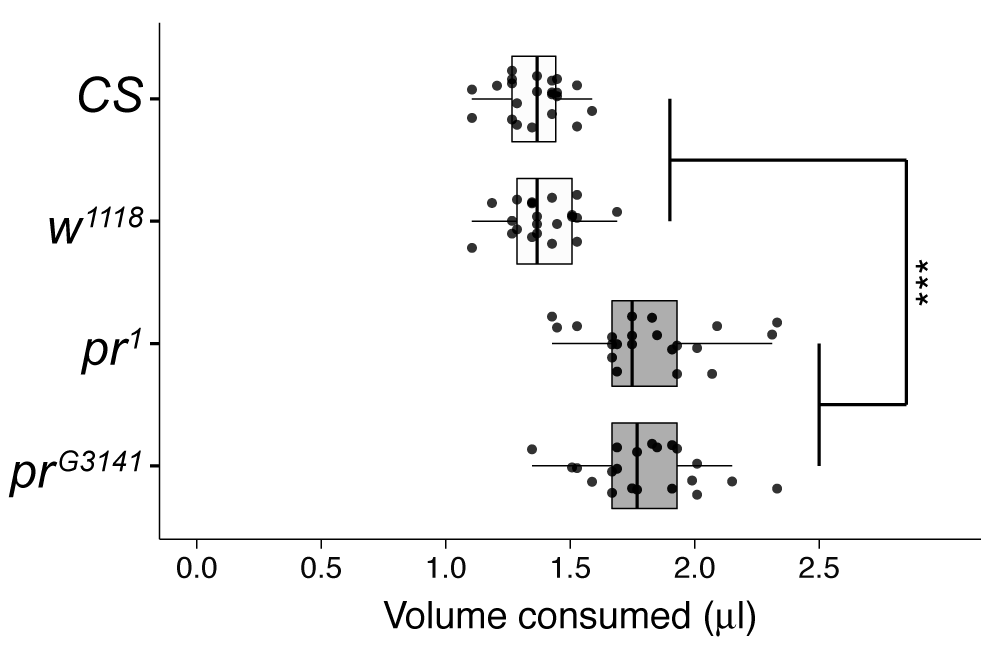

Supplement: S2 Fig — To determine whether pr also affects feeding in females, we measured ad libitum feeding in 5-day-old virgin females. Both prG3141 and pr1 (gray) eat more than the w1118 and Canton-S controls (white) (n = 21–22). Underlying numerical data for this figure can be found here: http://dx.doi.org/10.5061/dryad.8hm82. (TIF) [file pbio.2000532.s002.tif]

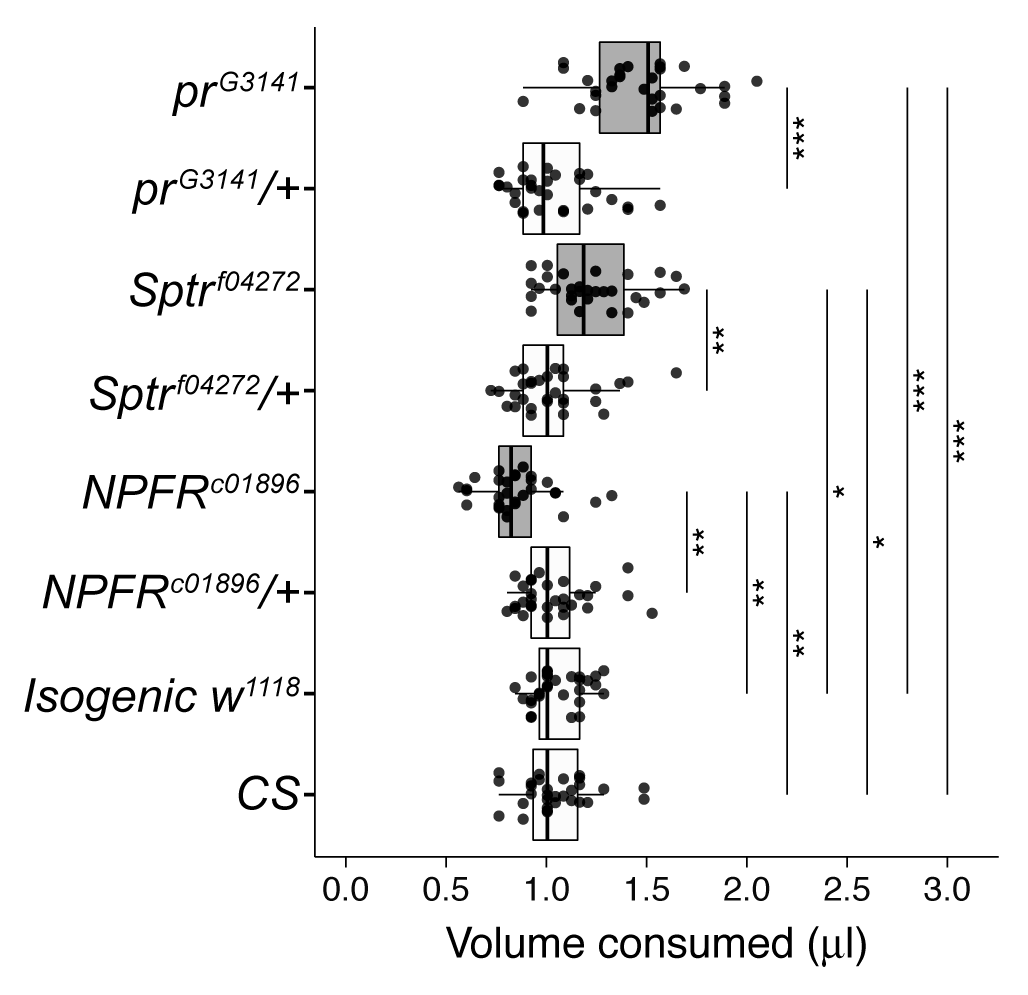

Supplement: S3 Fig — To assess the effect of genetic background on our feeding results, we back-crossed prG3141, Sptrf04272, and NPFRc01896 to an isogenized w1118 strain for seven generations and then measured their feeding behavior. The isogenized prG3141 and Sptrf04272 mutants showed increased feeding, while the NPFRc01896 mutant showed reduced feeding compared to the isogenic w1118 strain and Canton-S (n = 30). Underlying numerical data for this figure can be found here: http://dx.doi.org/10.5061/dryad.8hm82. (TIF) [file pbio.2000532.s003.tif]

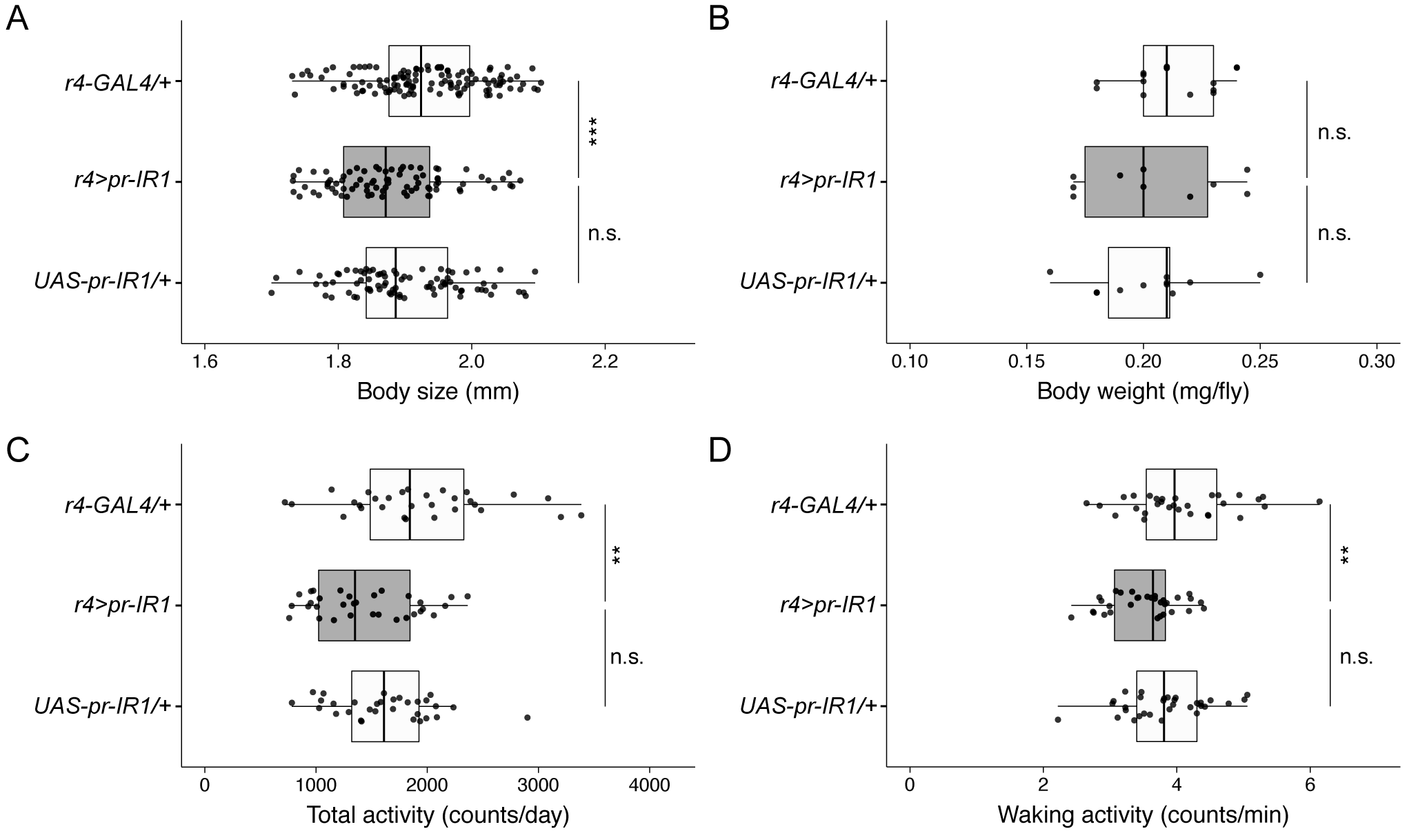

Supplement: S4 Fig — (A and B) Fat body-specific knock-down of pr does not significantly affect (A) body size (n = 81–117) or (B) dry weight (n = 10–15) as measured 1 day after eclosion. (C and D) Fat body-specific knock-down of pr does not significantly affect (C) total locomotor activity or (D) waking activity (n = 30–32). Underlying numerical data for this figure can be found here: http://dx.doi.org/10.5061/dryad.8hm82. (TIF) [file pbio.2000532.s004.tif]

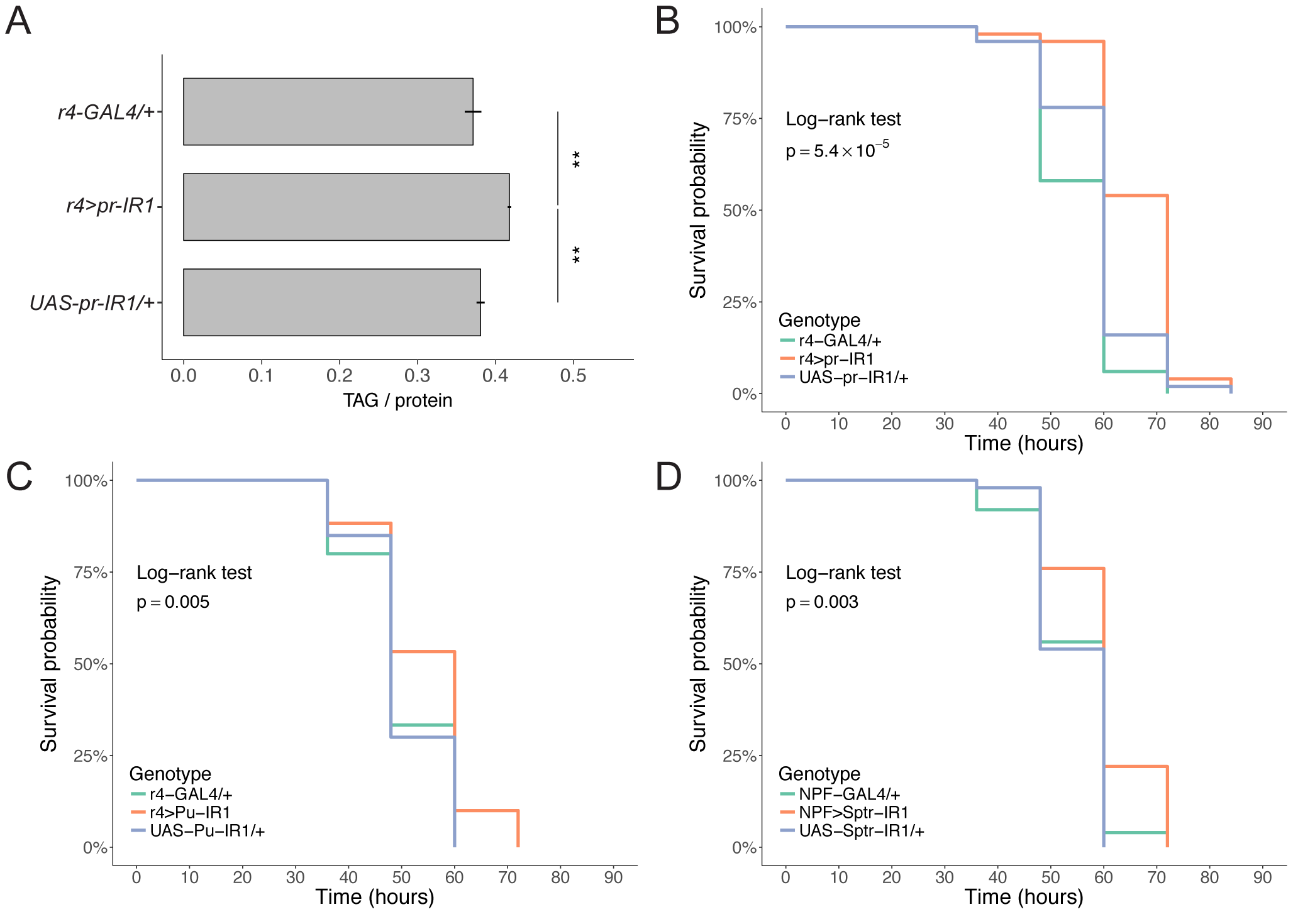

Supplement: S5 Fig — (A) Fat body-specific knock-down of pr induces a mild increase in whole body triacylglycerides (TAGs). TAG levels were normalized to protein levels (n = 5). (B) Fat body-specific knock-down of pr increases starvation resistance (n = 100). (C) Fat body-specific knock-down of Pu increases starvation resistance (n = 100). (D) NPF neuron-specific knock-down of Sptr increases starvation resistance (n = 100). For B–D, we made pair-wise comparisons of each heterozygous control line to the RNAi-expressing experimental line using the log-rank test. The reported p-value is the higher of these two values. Underlying numerical data for this figure can be found here: http://dx.doi.org/10.5061/dryad.8hm82. (TIF) [file pbio.2000532.s005.tif]

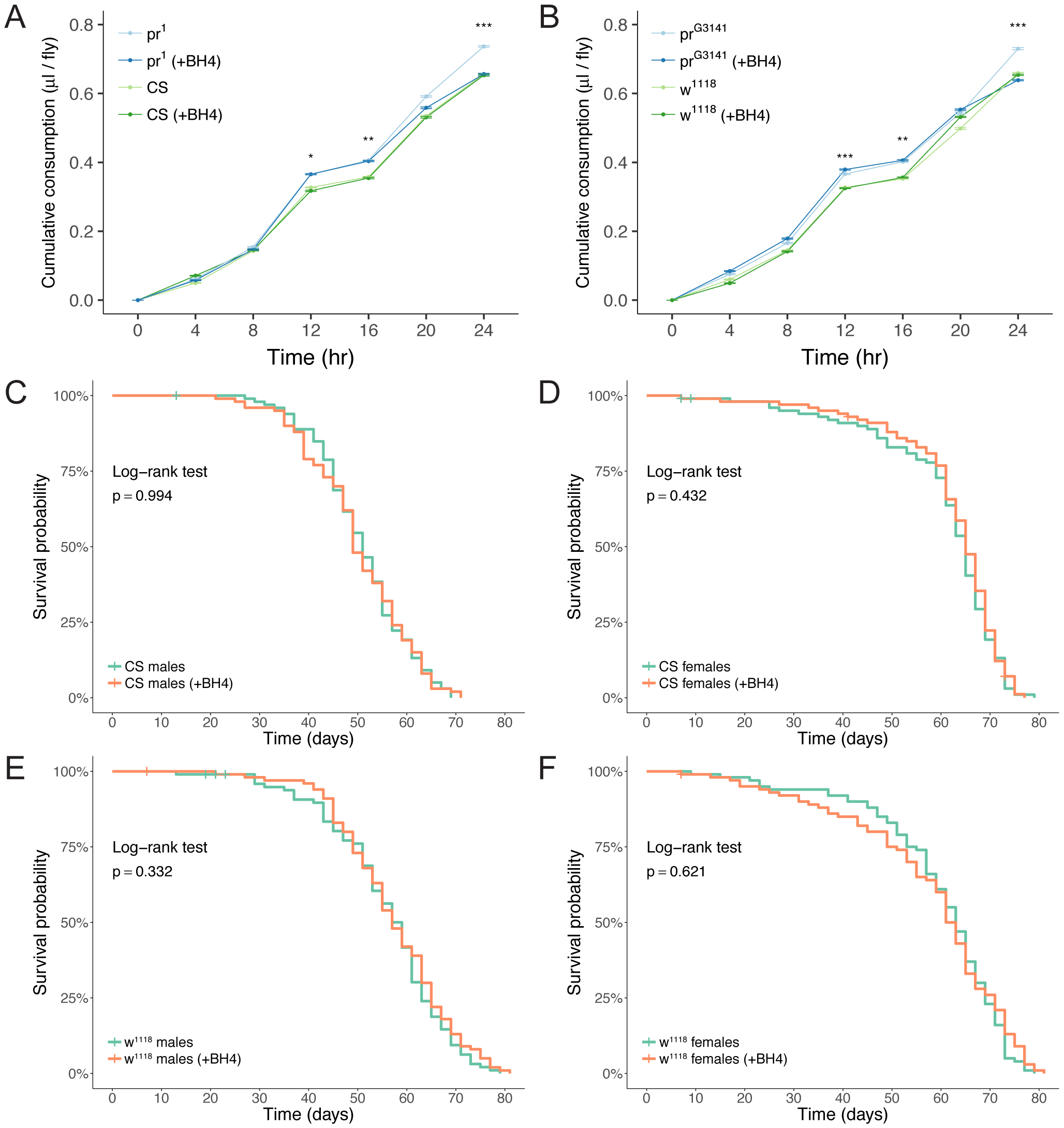

Supplement: S6 Fig — (A and B) Modified short-term CAFE assay measuring cumulative consumption every 4 hours for 24 hours. The hypomorphic pr mutants (i.e., pr1 and prG3141) eat significantly more than wild-type controls (i.e., w1118 and Canton-S) within 12 hr. Mixing a low dose of BH4 (0.17 mg/mL) into the feeding capillaries does not affect the consumption rates of the wild-type strains, but it rescues the increased feeding of the pr mutants within 16–20 hrs of feeding initiation (n = 27). (C–F) Continuous treatment with high dose BH4 (0.34 mg/mL) does not affect the lifespan of (C) Canton-S males, (D) Canton-S females, (E) w1118 males, or (F) w1118 females, suggesting BH4 is non-toxic. Underlying numerical data for this figure can be found here: http://dx.doi.org/10.5061/dryad.8hm82. (TIF) [file pbio.2000532.s006.tif]

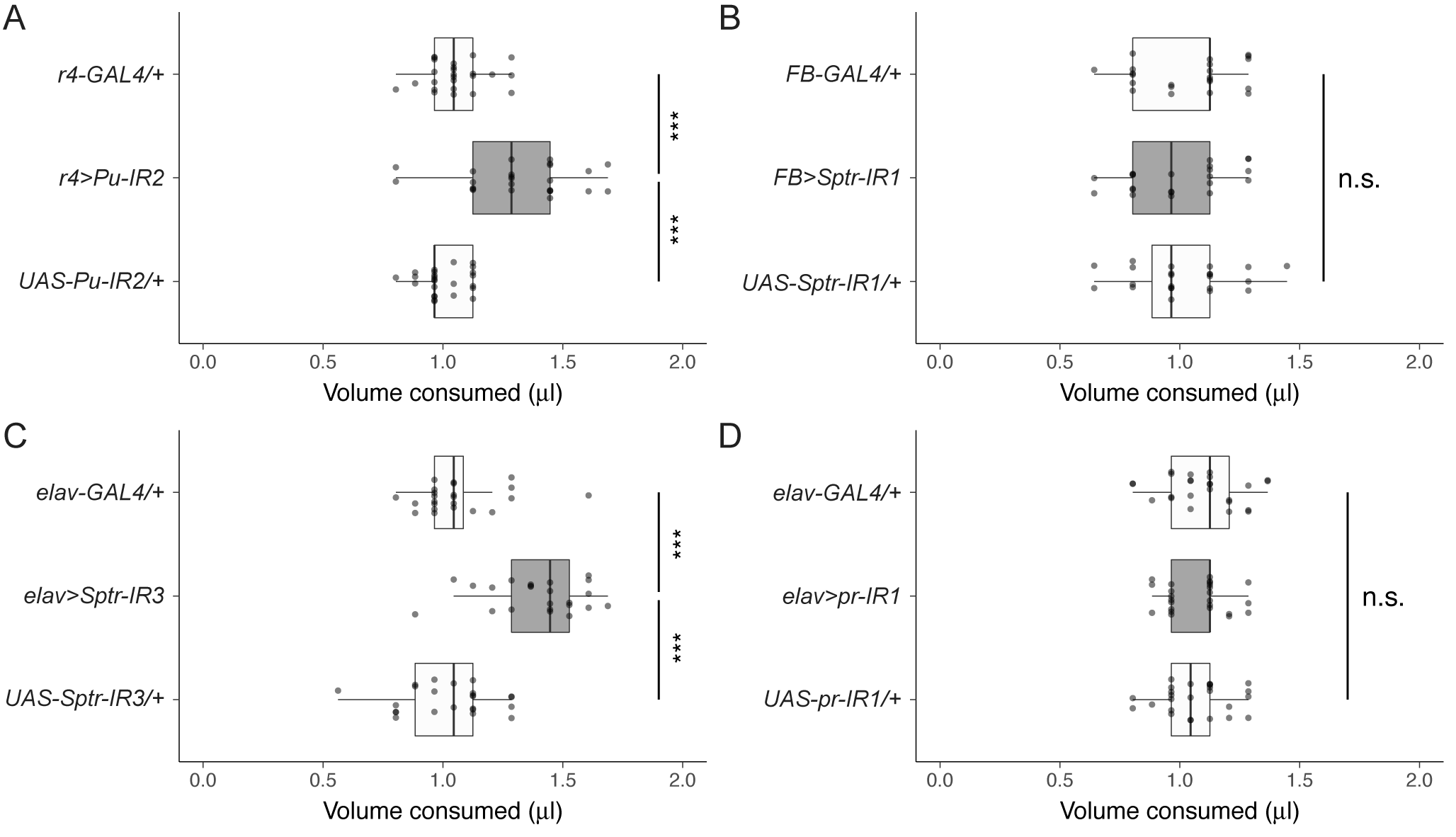

Supplement: S7 Fig — (A) Fat body-specific knock-down of Pu using a second RNAi line (UAS-Pu-IR2) increases feeding (n = 25). (B) Knock-down of Sptr using a second fat body GAL4 line (FB-Gal4) does not affect feeding (n = 22–23). (C) Pan-neuronal knock-down of Sptr using a second RNAi line (UAS-Sptr-IR3) increases feeding (n = 23). (D) Pan-neuronal knock-down of pr does not affect feeding (n = 25–26). Underlying numerical data for this figure can be found here: http://dx.doi.org/10.5061/dryad.8hm82. (TIF) [file pbio.2000532.s007.tif]

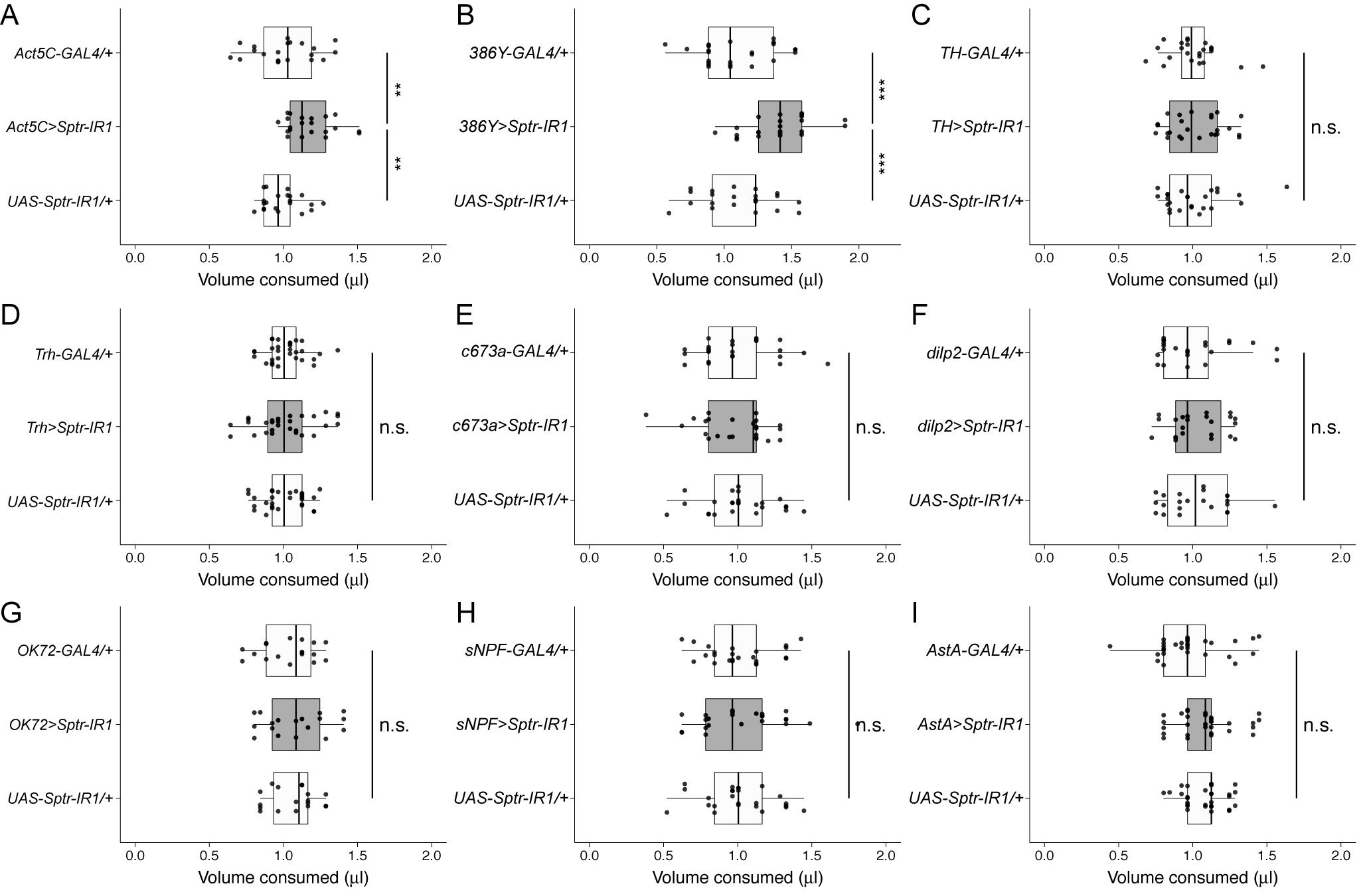

Supplement: S8 Fig — (A) Ubiquitous knock-down of Sptr using Act5c-GAL4 increases feeding (n = 21). (B) Knock-down of Sptr in most peptidergic neurons using the enhancer trap 386Y-GAL4 increases feeding (n = 23–25). (C) Knock-down of Sptr in dopaminergic neurons using TH-GAL4 does not affect feeding (n = 23–25). (D) Knock-down of Sptr in serotonergic neurons using Trh-GAL4 does not affect feeding (n = 30). (E) Knock-down of Sptr in “obesity blocking neurons” using the enhancer trap c673-GAL4 does not affect feeding (n = 25). (F) Knock-down of Sptr in the insulin producing cells using dilp2-GAL4 does not affect feeding (n = 22–23). (G) Knock-down of Sptr in oenocytes using OK72-GAL4 does not affect feeding (n = 18–19). (H) Knock-down of Sptr in short neuropeptide F neurons using sNPF-GAL4 does not affect feeding (n = 18–19). (I) Knock-down of Sptr in allatostatin-A neurons using AstA-GAL4 does not affect feeding (n = 24–25). Underlying numerical data for this figure can be found here: http://dx.doi.org/10.5061/dryad.8hm82. (TIF) [file pbio.2000532.s008.tif]

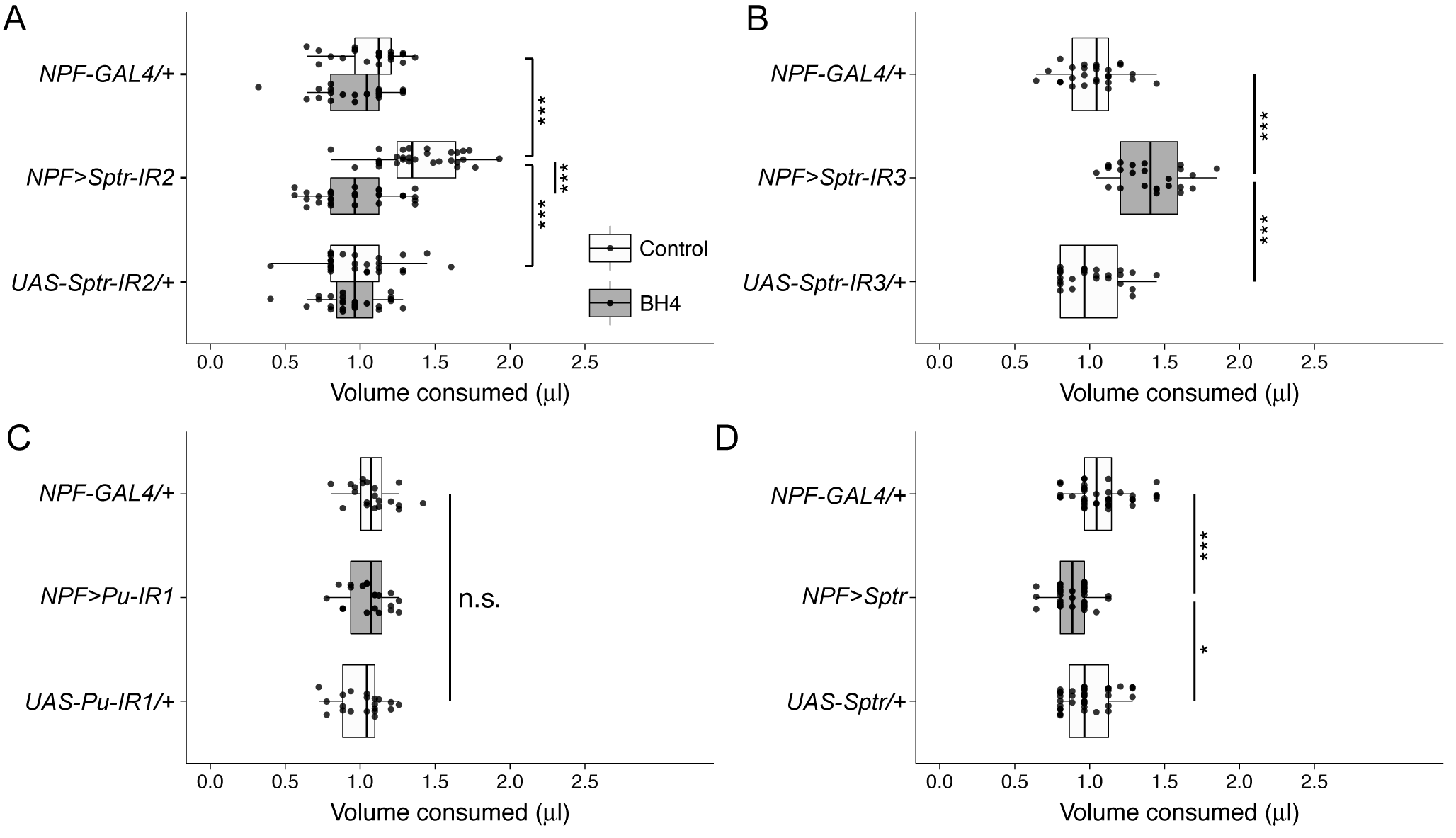

Supplement: S9 Fig — (A) NPF neuron-specific knock-down of Sptr using a second RNAi line (UAS-Sptr-IR2) induces a hyperphagic phenotype that is rescued by pre-feeding the flies with 0.17 mg/ml BH4 (n = 24–30). (B) NPF neuron-specific knock-down of Sptr with a third RNAi line (UAS-Sptr-IR3) increases feeding (n = 26–27). (C) NPF neuron-specific knock-down of Pu does not affect feeding (n = 20). (D) NPF neuron-specific over-expression of Sptr reduces feeding (n = 40). Underlying numerical data for this figure can be found here: http://dx.doi.org/10.5061/dryad.8hm82. (TIF) [file pbio.2000532.s009.tif]

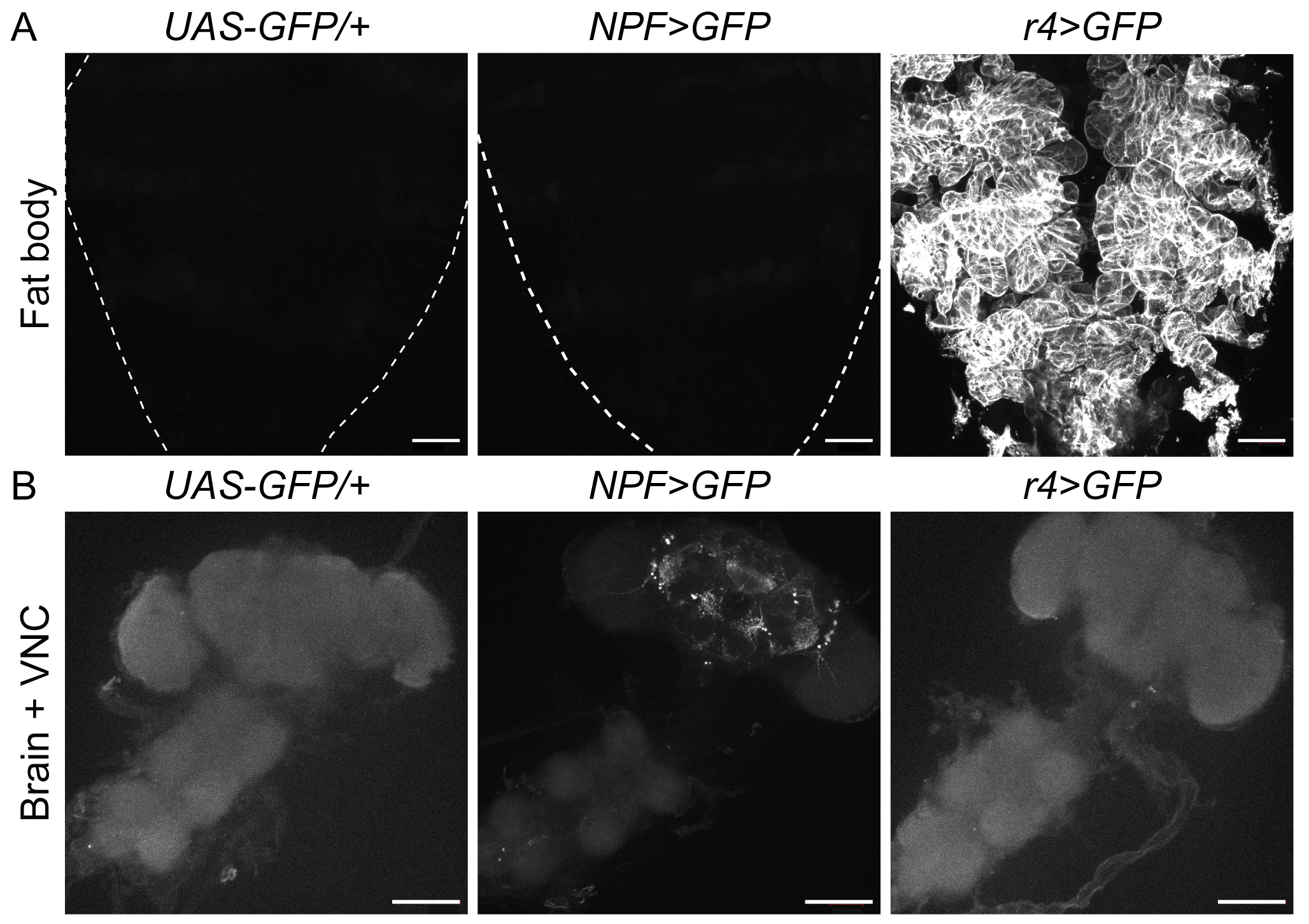

Supplement: S10 Fig — (A) Staining of the abdominal fat body with a GFP-specific antibody shows that r4-GAL4 but not NPF-GAL4 drives expression in the fat body. Dotted lines mark the edges of the abdomen. Scale bars = 100 μm. (B) Staining of the adult brain and ventral nerve cord with a GFP-specific antibody shows that NPF-GAL4 but not r4-GAL4 drives expression in the brain. Scale bars = 100 μm. (TIF) [file pbio.2000532.s010.tif]

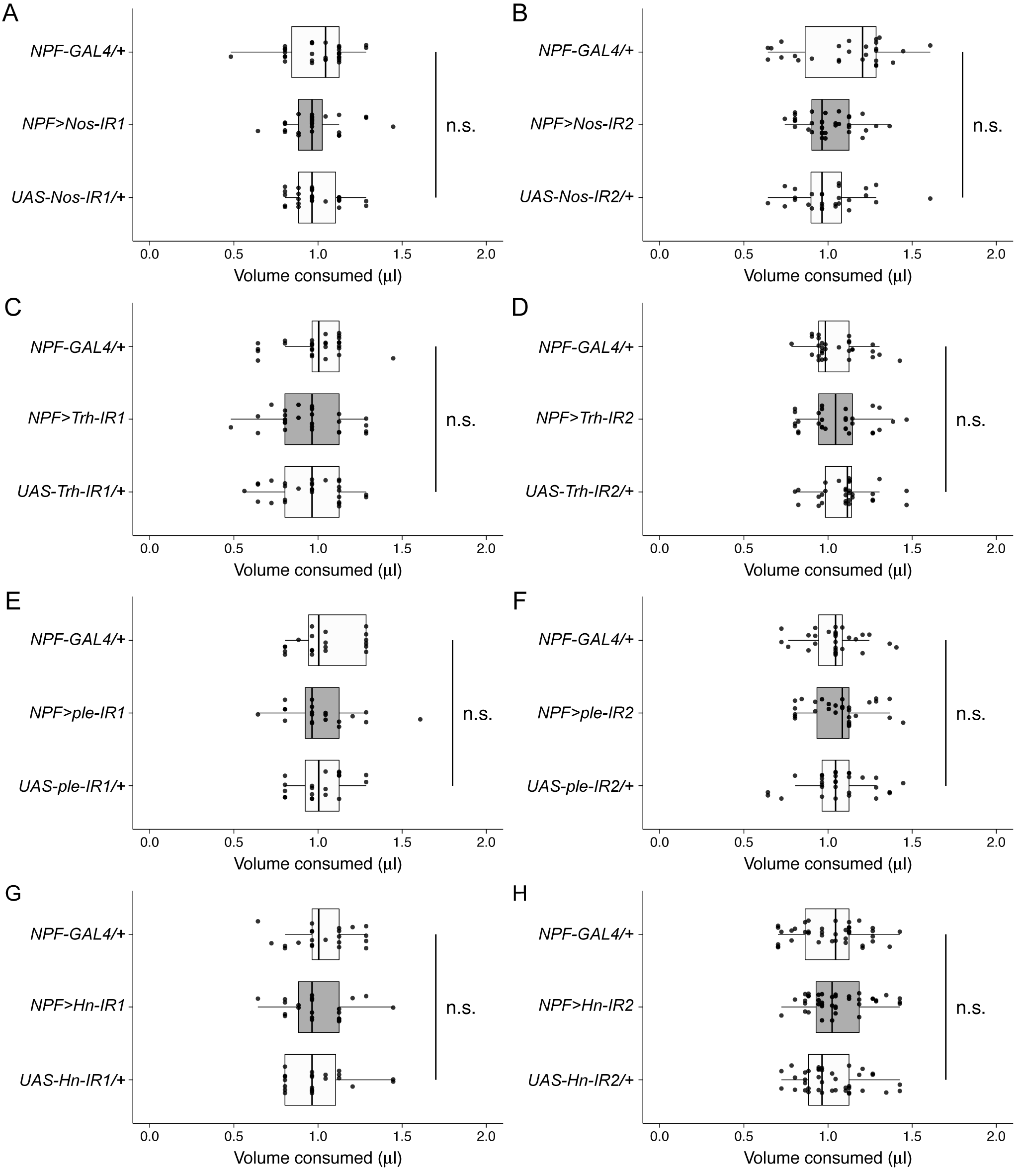

Supplement: S11 Fig — (A and B) Nitric oxide synthase (A, n = 30; B, n = 27–35). (C and D) Tryptophan hydroxylase (C, n = 28; D, n = 30). (E and F), pale (Tyrosine hydroxylase) (E, n = 20; F, n = 30). (G and H), Henna (Phenylalanine hydroxylase) (G, n = 22; H, n = 40). Underlying numerical data for this figure can be found here: http://dx.doi.org/10.5061/dryad.8hm82 (TIF) [file pbio.2000532.s011.tif]

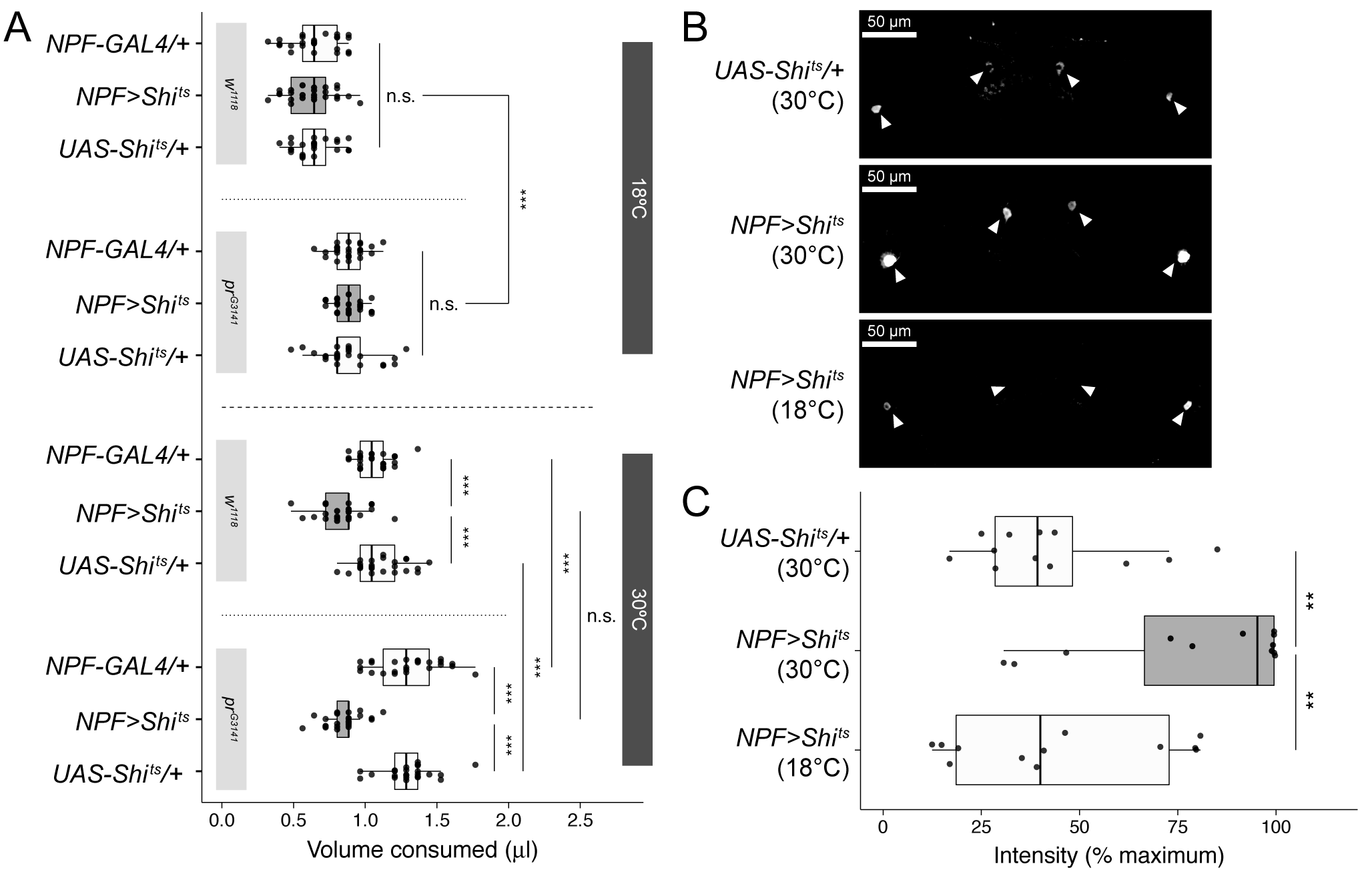

Supplement: S12 Fig — (A) Conditional inhibition of the NPF neurons with temperature-sensitive Shibire (Shits). At the permissive temperature (18°C), flies with the prG3141 mutant background eat more than those with the w1118 background (n = 25). Inhibition of the NPF neurons at 30°C reduces the feeding of flies with both the prG3141 and w1118 backgrounds (n = 25), confirming Purple functions upstream of the NPF neurons. (B) Staining of dissected brains with an NPF-specific antibody. Inhibition of the NPF neurons at 30°C (center) increases NPF accumulation in the NPF neuron cell bodies compared to controls. Arrowheads indicate NPF neuron cell bodies. All brains were imaged with the same confocal settings. (C) Boxplots comparing the NPF signal intensities of 12 stained cell bodies from 3 brains for each condition. Inhibition of the NPF neurons presumably increases the accumulation of NPF by blocking its release. Underlying numerical data for this figure can be found here: http://dx.doi.org/10.5061/dryad.8hm82 (TIF) [file pbio.2000532.s012.tif]
